# Not All Rules Are Equal: Rare Conditional Rules Shape Behaviour but Yield to Global Probability in Passive Listening

**DOI:** 10.1111/ejn.70500

**Published:** 2026-04-20

**Authors:** Nina Coy, Alexandra Bendixen, Annika Löhr, Sabine Grimm, Erich Schröger, Urte Roeber

**Affiliations:** ^1^ Cognitive Systems Lab, Institute of Physics Chemnitz University of Technology Chemnitz Germany; ^2^ Max Planck School of Cognition Leipzig Germany; ^3^ Wilhelm‐Wundt Institute for Psychology Leipzig University Leipzig Germany; ^4^ Physics of Cognition Lab, Institute of Physics Chemnitz University of Technology Chemnitz Germany

**Keywords:** auditory prediction, EEG ERP, EFA/PCA, MMN, sensory systems

## Abstract

The human auditory system rapidly encodes auditory regularities. Evidence comes from the oddball paradigm, in which frequent (standard) sounds are occasionally replaced with a rare (deviant) sound. Deviants relative to standards typically elicit signs of prediction error (e.g., MMN and P3a). It is, however, less clear whether deviants, which also bear predictive information but are encountered less often than standards, might inform auditory prediction. To investigate this, naïve participants listened to sound sequences constructed according to a new, modified version of the oddball paradigm: two kinds of deviants differing in their probability of repetition yield the sound actually following a deviant either conditionally likely or unlikely. As this sound is either the same deviant (repetition) or a standard (no repetition), it is either unlikely or likely with respect to the global stimulus probability at the same time. In an active deviant detection task, we replicated previous behavioural findings, demonstrating that predictive information carried by deviants (conditional probability) is extracted when behaviourally relevant. Our analyses further reveal that respective response time effects increase over the course of the task. However, in a passive listening setting, both MMN and P3a were confined to violations of rules based on global probability, while not being sensitive to conditional probability. Though some sensitivity to conditional probability had been observed in a previous study, these effects were tiny compared to those of global probability. Thus, the auditory system seems to mainly rely on rules that are encountered frequently (standard regularity), at least during passive listening.

## Graphical abstract

The auditory system rapidly detects violations of sound regularities, but it remains unclear whether rarely encountered sounds violating such established regularities (i.e., deviants) themselves can inform prediction. In an adapted oddball paradigm, behavioural results show that conditional rules associated with rarely encountered deviants can be extracted when task‐relevant, whereas brain activity during passive processing was shaped by global probabilities. Thus, the auditory system seems to mainly rely on rules that are encountered frequently (standard regularity), at least during passive listening.

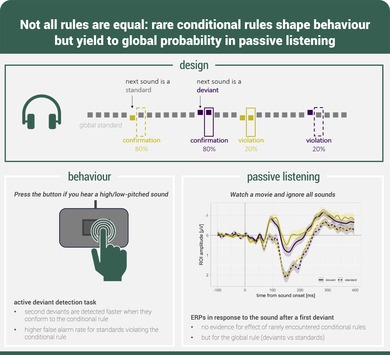

**Abbreviations**
BF: Bayes factor
CI: credible interval
DC: direct current
EEG: electroencephalography
EFA: exploratory factor analysis
EOG: electrooculography
ERP: event‐related potential
FIR‐filter: finite impulse response filter
IC: independent component
ICA: independent component analysis
LOO: leave‐one‐out
LOOIC: leave‐one‐out information criterion
MMN: mismatch negativity
PP: posterior probability
RT: response time
SOA: stimulus onset asynchrony

## 1 Introduction

The human brain is sensitive to recurring sensory information (Barascud et al. 2016; Garrido et al. 2009; Kaernbach 2004; Näätänen et al. 2007). It has been suggested that regular relationships governing how event sequences unfold can be used to infer predictions about future events (Friston 2005; Näätänen et al. 2001; Winkler 2007). Yet, even in the simplified stimulation sequences used in laboratory settings, there are more potential rules than typically accounted for (Nousak et al. 1996; Sams et al. 1984; Schröger et al. 2023; Winkler 2007). One example is the classic oddball paradigm, in which a frequent standard stimulus is occasionally replaced by a rare deviant stimulus. Due to widely applied randomisation constraints in the design, each deviant is followed by a standard sound with 100% probability, making the deviant a more precise predictor for the subsequent standard than any standard in the sequence (which is typically followed by a standard in only 80%–90% of the time). The recurrence of the standard is typically defined as the regularity governing the sequence, whereas the occurrence of the deviant serves as the vehicle to probe the extraction of that standard regularity. When the observed brain responses differ between rule‐conforming standard and rule‐violating deviant, it is inferred that the standard regularity was successfully extracted (e.g., Fitzgerald and Todd 2020; Garrido et al. 2009; Winkler 2007). Initially, the paradigm was based on rather simple regularities (i.e., the standard regularity is defined by an exact repetition). Later studies revealed that much more complex relationships between standard stimuli can be extracted by the human auditory system (Bendixen et al. 2008; Horváth et al. 2001; Paavilainen 2013; Tervaniemi et al. 1994; Tsogli et al. 2019; van Zuijen et al. 2006). Yet one important factor is common to all standard regularities: The rule in question is encountered frequently. Furthermore, it is well established that the representation of a standard rule strengthens with an increasing number of rule encounters (Baldeweg et al. 2004; Bendixen et al. 2007; Haenschel et al. 2005). In contrast, it is not well understood yet under which conditions and to what degree predictive information carried by the rarely encountered deviant might also inform auditory processing.

The idea of considering deviants as predictors in their own right has been suggested before (Todd and Mullens 2011; Todd and Robinson 2010) but has not received much attention so far: first, due to their characteristic role as test probes for the standard regularity and, second, due to the widespread assumption in mismatch negativity (MMN) research that the return to the standard regularity (i.e., the first standard after a deviant event) constitutes a regularity violation as well (Winkler 2007). The latter implies that the transition from deviant to standard is not predicted (despite its certainty in terms of conditional probability). MMN is an event‐related‐potential (ERP) component prominently associated with prediction error processing (Fitzgerald and Todd 2020; Friston 2005; Garrido et al. 2009). It is typically observed as a frontocentrally distributed negative deflection with polarity inversion at the mastoids peaking around 100–250 ms in the deviant‐minus‐standard difference wave (Ford et al. 1976; Näätänen et al. 1978). As we have described in more detail in a previous study (Coy et al. 2024), there exist only few studies that have investigated the deviant‐to‐standard transition (Koistinen et al. 2012; Nousak et al. 1996; Roeber et al. 2003, 2005; Sams et al. 1984). Interestingly, instead of a modulation of MMN, some studies (Koistinen et al. 2012; Roeber et al. 2003, 2005) report modulations in the form of P3a‐like activity, which indicates the potential involvement of higher processing levels. P3a is typically observed as a frontocentral positivity in the ERP peaking in the range of 300–500 ms after deviation onset (Escera et al. 2000; Polich 2007). P3a is commonly associated with the (involuntary) attentional orienting towards the deviant (Berti et al. 2004; Escera et al. 2000; Polich 2007), though it was also related to stimulus evaluation (Friedman et al. 2001; Horváth et al. 2008) and stimulus selection at the level of working memory (Dien et al. 2004). Unfortunately, none of the studies investigating deviant‐to‐standard transitions included an appropriate control condition for effects related to the potential conditional prediction carried by the deviant. One issue is that in the classical oddball paradigm, the conditional rule associated with the deviant (*the next sound will be a standard*) and the global rule associated with the standard (*the most probable sound in any trial is the standard*) yield the same prediction for the sound following a deviant event (*prediction of a standard*). Thus, a dissociation is not possible. The second issue is related to sequential effects when the standard following a deviant is compared against a standard following a standard (also referred to as the global standard). There is likely a different amount of stimulus‐specific adaptation in a standard‐deviant‐*standard* compared to standard‐standard‐*standard* microsequences (Ulanovsky et al. 2004). Also, a deviant might transiently weaken the global prediction (since it recently failed), or there might be other carryover effects from the preceding deviant ERP, especially in fast‐paced protocols.

In a range of studies (Müller et al. 2005a, 2005b; Rosburg et al. 2018; Todd and Mullens 2011; Todd and Robinson 2010), a different approach was used in which deviants repeat with a certain probability before returning to the standard regularity, to assess whether deviants might be used to predict the subsequent stimulus. Typically, the response to such a second deviant is compared against the response to the first deviant. But again, due to the differences in immediate stimulus history (standard‐deviant‐*deviant* vs. standard‐standard‐*deviant*), there might be differential stimulus‐specific adaptation or differential responsiveness of the deviant detection system (Horváth et al. 2008; Rosburg et al. 2018).

**FIGURE 1:**
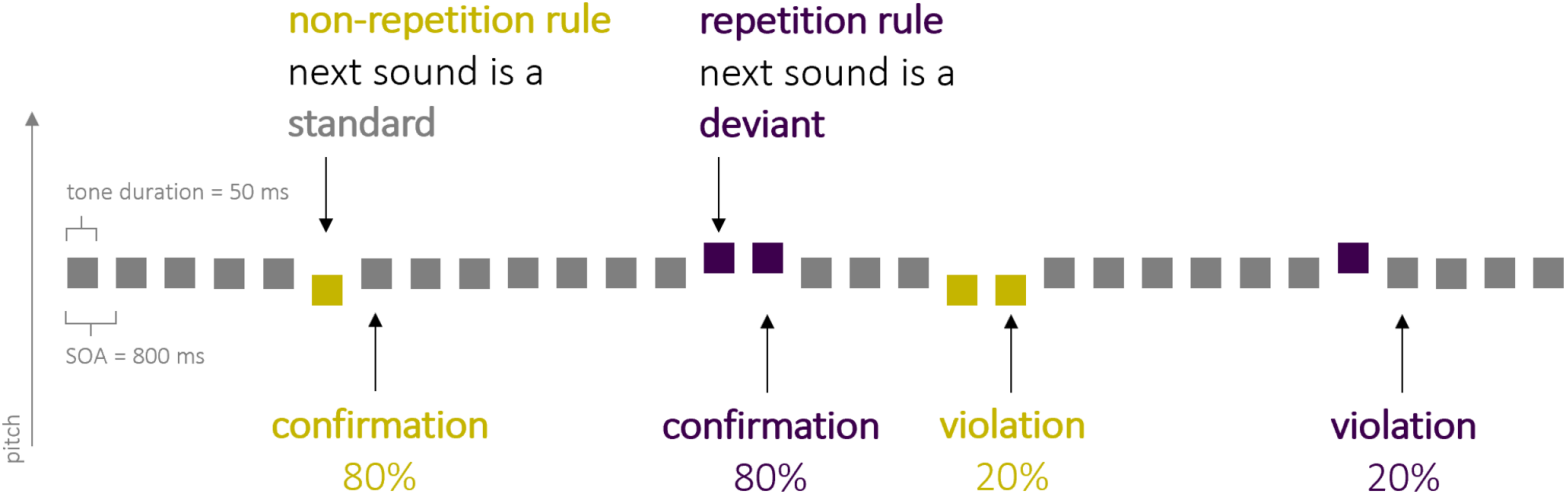
Experimental design illustrated in an exemplary stimulus sequence. The frequently presented standard sound (440 Hz) was occasionally replaced by one of two pitch deviants. For half of the participants, the high‐pitch deviant (547.37 Hz) was associated with high repetition probability (purple; repetition rule) and the low‐pitch deviant (349.23 Hz) with low repetition probability (yellow; nonrepetition rule); for the other half of the participants, this association was reversed. Each deviant type could occur with equal probability among the standards.

To address the issue of microsequence effects (i.e., local stimulus history), we recently proposed a variation of the classic oddball paradigm, which contrasts different repetition probabilities within an ongoing stimulation (Coy et al. 2022): Two diametrically opposed deviant repetition rules are consistently associated with two deviants of differing pitch throughout the experiment (Figure 1)—one deviant is more likely followed by an identical second deviant than by a standard (repetition rule: high repetition probability) and the other deviant is more likely followed by a standard than by an identical second deviant (nonrepetition rule: low repetition probability). Note, no deviant is ever followed by a nonidentical deviant.

These repetition rules differentially affected performance in a deviant detection task (Coy et al. 2022, 2024), although participants were not informed about their existence: response times decreased from first to second deviant when deviant repetition probability was high but not when it was low. Although there was no effect of repetition rule on the hit rates (possibly because they were near ceiling), false‐alarm rates increased by a factor of 3 in response to standards following single‐deviant presentations for high compared to low deviant repetition probability. These findings suggest that deviants can be used to infer precise expectations about auditory events in the imminent future, at least when deviants are explicitly relevant to the current task.

Differential processing of standard and deviant stimuli is observable in the classic oddball paradigm even when participants are instructed to ignore (or to not actively attend) these stimuli; that is, building and maintaining internal regularity representations appear to not require direct attention or intention (Garrido et al. 2009; Näätänen et al. 2007; Schröger 2005; Sussman 2007; Sussman et al. 2014). In a previous ERP study (Coy et al. 2024), we thus applied our newly developed paradigm (Coy et al. 2022) to investigate whether the conditional repetition rules associated with the deviants are extracted by the auditory system when all sounds are to be ignored. If ERPs in response to the stimuli directly following a first deviant (standard or deviant, respectively) differ as a function of deviant repetition probability in a passive‐listening task, this would provide substantial evidence to the notion that deviants inform prediction. However, we found moderate evidence against MMN and P3a elicitation when a second deviant was conditionally unpredictable compared to predictable. Yet we observed a slow‐wave positivity in response to unpredictable compared to predictable standards following a first deviant. At the MMN level, evidence was inconclusive, but there was moderate evidence in favour of P3a elicitation for conditional rule violations relative to confirmations. This suggests that rarely encountered deviants probably do not drive auditory sensory prediction but they might inform processing at higher levels.

In the previous study (Coy et al. 2024), we used a jittered stimulus onset asynchrony (SOA; uniform distribution 550–700 ms), because it was not entirely clear whether the stimulation rate and the passive‐listening setting of that study could result in the perceptual integration of two deviant tones into one deviant event (Sussman and Winkler 2001). This concern turned out to be unsubstantiated, as we observed distinct and separate ERPs in response to successive deviants. In the current study, we opted for a fixed SOA, as a jittered SOA has some drawbacks: SOA has a strong effect on amplitudes by itself (Jaffe‐Dax et al. 2017; Pereira et al. 2014; Rosburg et al. 2010; Sams et al. 1993), thus likely increasing the variability in ERP components' amplitudes. Additionally, we decided to increase the temporal separation between sounds (800 ms SOA), as processing appeared to span into the subsequent trial for the shorter SOAs in the previous study.

The main aim of the current study is to probe whether the findings from the previous study (Coy et al. 2024) can be observed in a different sample, while improving the reliability of the amplitude estimates (less variability from SOAs and less temporal overlap of ERPs). Specifically, we address the following research questions: (1) Does the auditory system learn to predict a second deviant (based on conditional probability) even though this stimulus violates the global rule (defined by base probability), and (2) does the auditory system process a standard stimulus as a mismatch to a local rule (conditional probability) although the stimulus is in agreement with the global rule (base rate)? Furthermore, we are interested in whether (3) the ERPs in response to a sound following a deviant are driven either or both by the global rule (frequent standard vs. rare deviant) and the conditional rule associated with the deviants.

To address Questions 1 and 2, we look at the level of behaviour (accuracy and response times) during active deviant detection, and we examine the ERP response elicited by the stimuli following a first deviant—either a deviant (1) or a standard (2)—during passive listening with a focus on MMN and P3a activity as electrophysiological indicators of prediction error processing. In our variation of the oddball paradigm, it is possible to contrast between *rule violation* and *rule confirmation* for each conditional deviant repetition rule. More specifically, both deviant after deviant and standard after deviant can be compared as a function of deviant repetition probability, respectively. In the case of deviant repetition (deviant after deviant), the second deviant constitutes a rule violation in the context of a low repetition probability (nonrepetition rule) and a rule confirmation in the context of a high repetition probability (repetition rule). The relevant difference is thus defined as low repetition probability (rule violation) minus high repetition probability (rule confirmation). In the case of nonrepetition (standard after deviant), the standard following a deviant associated with high repetition probability is the rule violation, whereas the standard following a deviant associated with low repetition probability is the rule confirmation. The relevant difference here is thus defined as high repetition probability (rule violation) minus low repetition probability (rule confirmation). In this way, the paradigm allows also to dissociate conditional probability (*what is the most likely stimulus given the preceding deviant*) from global probability (*deviants are overall less probable than standards*).

Like in the preceding study (Coy et al. 2024), we use temporal exploratory factor analysis (EFA) to decompose the ERP data into a set of underlying factors corresponding to its constituent components. Visually identified peaks may be a suboptimal indicator of underlying brain signals, because the strong temporal and spatial overlap of ERP components results in potentially biassed estimates of amplitudes, latencies or topographies (Scharf et al. 2022). At the ERP level, we refer to the characteristic negative difference between deviants and standards within the MMN time range as *observed MMN* and to the respective positive difference in the P3a time range as *observed P3a*. We will first identify and label the factors obtained via EFA at the stimulus level (e.g., N1, P2 and N2) in the typical MMN and P3a time ranges. We use the factors thus identified to probe whether the predictive information carried by deviants (high vs. low conditional probability of repetition) is extracted, while controlling for global stimulus probability through the design. Specifically, if the respective difference between violation and confirmation of a conditional rule (as described above) is negative within the MMN time range, this will be interpreted as evidence for the extraction of rare conditional rules at the sensory level, a corresponding positive difference within the P3a time range as evidence for their representation at higher processing levels. Relating to Research Question 3, differences (negative in MMN and positive in P3a time range) between deviant (global rule violated) and standard (global rule confirmed) stimuli following a first deviant (comparable stimulus history) are taken as an indicator for the presence of predictions inferred from the global rule.

In the active deviant detection task, we expect the predictive information provided by the deviants to be reflected in behaviour. When the prediction generated upon encountering the first deviant is matched by the incoming sensory input for the next sound event (prediction confirmation), processing should be facilitated. When the prediction generated upon encountering the first deviant is mismatched by the incoming sensory input for the next sound event (prediction error), processing should be impeded. To specify, when a first deviant is followed by a second deviant, response times or error (miss) rates should be lower for high repetition probability (rule confirmation) than for low repetition probability (rule violation). When a first deviant is followed by a standard, error (false alarm) rates should be lower for low repetition probability (rule confirmation) than for high repetition probability (rule violation). Additionally, we are interested in whether the differential response time facilitation (i.e., larger RT facilitation for high compared to low deviant repetition probability) observed in both previous studies (Coy et al. 2022, 2024) is conditional on time of exposure. Participants will already have been exposed to the stimulation sequences for about 90 min (passive‐listening task) before conducting the active task. Thus, we want to probe whether the differential RT facilitation between the conditional rules observed in the previous studies (Coy et al. 2022, 2024) is already present in the beginning of the active task, or whether it will only emerge after some time, and whether it will increase with time.

## 2 Methods

### 2.1 Participants

Data were collected from 60 participants (*M* = 22.31 years, ranging from 19–33 years; 50 women, 8 men and 2 nonbinary), who all reported normal hearing, normal or corrected‐to‐normal vision, no history of neurological conditions nor intake of any related prescribed drugs affecting the central nervous system. One participant was excluded from the behavioural analysis, because they reported misunderstanding the task instruction. Participants received compensation either in form of payment (12€/h) or course credit. Protocols and procedures were in accordance with the Declaration of Helsinki and approved by the Ethics Advisory Board at Leipzig University (RF: 2022.02.22_eb_134). The sample size was predetermined following the fixed‐n approach for planning informative Bayes Factor (*BF*) designs (Schönbrodt et al. 2017), such that (a) there is at least an 80% probability of detecting a true effect with a *BF*_10_ evidence threshold of 6 (moderate evidence) when the population effect is 0.5 (medium‐sized); (b) there is less than a 0.5% probability of falsely rejecting the null hypothesis with a *BF*_01_ evidence threshold of 1/6. Unlike in classical significance testing, even if the desired evidence threshold is not reached with a given sample size, the *BF* is still interpretable (see Section 2.8) and distinguishes between inconclusive evidence and evidence for the null hypothesis.

### 2.2 Procedure and Apparatus

Participants were seated comfortably in a soundproof chamber during the experiment. Experimental stimulation was delivered using Octave (Eaton et al. 2021; on Linux) and Psychtoolbox‐3 (Kleiner et al. 2007). Auditory stimuli were presented at an A‐weighted sound pressure level of approximately 60 dB (A) via headphones (HD25‐1 II 70 Ω, Sennheiser GmbH Co. KG, Germany). During the main part of the experiment (recording of EEG), participants were instructed to watch a silent movie with subtitles while ignoring the sounds (passive‐listening task). All visual stimulation was displayed on a 24″ LCD display (ViewPixx/EEG, VPixx Technologies Inc., Canada, resolution 1920 × 1080 pixels, refresh rate of 120 Hz), which was placed at a comfortable viewing distance in front of the participant (∼60 cm). A behavioural control task was performed after the EEG session, in which participants were asked to actively detect deviants while not being informed about the deviant repetition rules. Feedback on performance was given after each block (mean reaction time, hit and false‐alarm rates). During this control task, participants were asked to gaze at a white fixation cross on a black screen. Behavioural responses (button press) were registered via the Response Time Box (Li et al. 2010).

### 2.3 Stimuli and Design

The experimental design and stimuli were the same as in the study by Coy et al. (2024), except for explicitly counterbalancing the deviant type encountered first between experimental blocks and for the stimulation speed: Stimulus onset asynchrony (SOA) was fixed at 800 ms (instead of jittered between 550 and 700 ms). Participants were presented with sound sequences that were constructed based on a recently proposed variation of the classic oddball paradigm (Coy et al. 2022), systematically manipulating conditional deviant repetition probability (Figure 1). Specifically, a medium‐pitched sine tone (440 Hz) served as the frequent standard, and a higher (547.37 Hz) and a lower (349.23 Hz) pitched sound occurred as rare deviants that, crucially, followed diametrically opposed repetition rules: Whereas one deviant type was mostly followed by a standard sound (low repetition probability and nonrepetition‐rule deviant), the other deviant type was mostly followed by an identical deviant sound (high repetition probability and repetition‐rule deviant). Occasionally though (20% of the trials), these deviant repetition rules were violated: The low‐repetition‐probability deviant repeated, the high‐repetition‐probability deviant was directly followed by a standard. The association of a deviant type (pitch) with a repetition rule was counterbalanced across participants. Each block consisted of 435 trials—345 standards and 90 deviants. Across the 20 blocks, there were altogether 6900 standards and 1800 deviants. Each of the two deviants—associated with the high or low repetition probability—was presented in 600 trials as a first deviant (following a standard) in order to balance the amount of exposure with each transitional regularity. As a consequence, high‐repetition‐probability deviants (1080 trials; first and second deviants together) occurred 1.5 times more often than low‐repetition‐probability deviants (720 trials). For each deviant‐repetition rule, there were 480 rule‐conforming trials and 120 rule‐violating trials. The behavioural control task consisted of six blocks with 220 trials each.

### 2.4 EEG Recording

The electroencephalogram (EEG) was recorded at a 512‐Hz sampling rate, using a 64 channel BioSemi ActiveTwo system with a DC amplifier and ActiView (BioSemi). Active Ag/AgCl‐electrodes were mounted in a suitable head cap on the scalp in accordance with the 10–10 extension of the international 10–20 system (Chatrian et al. 1985). Two external electrodes were placed at the mastoids, one electrode on the tip of the nose to serve as reference (offline). Additionally, horizontal and vertical electrooculograms (EOGs) were recorded with two electrodes placed on the outer canthi of the eyes, and above and below the right eye, respectively.

### 2.5 EEG Preprocessing

#### 2.5.1 Main Data Set

EEG analysis was implemented in MATLAB R2024b with the EEGLAB toolbox (Delorme and Makeig 2004, Version 2024.0). The signal was filtered offline with a 0.1‐Hz high‐pass filter (Hamming windowed sinc FIR filter, order = 8448, transition band width = 0.2 Hz). Independent component analysis (ICA) was used to identify and remove activity related to ocular artefacts (eye blinks, horizontal and vertical eye movements and presaccadic spikes) from the recorded signal. A separate copy of the data (ICA data copy) was preprocessed specifically to improve IC decomposition (see the next section for details). The demixing matrix resulting from ICA was transferred onto the original EEG data. The *dipfit 2.3* plugin was used to fit a single equivalent current dipole for each independent component and then passed to the *fitTwoDipole* plugin (Piazza et al. 2016) to identify symmetrically constrained dipoles. This was done to improve the subsequent classification of the independent components by the *ICLabel* plugin (Pion‐Tonachini et al. 2019). All components exceeding a 70% probability threshold of being of ocular origin while maintaining a probability of being brain activity below 5% were removed from the data (*M* = 3.60 components per participant). Subsequently, the signal was filtered with a 45‐Hz low‐pass filter (Hamming windowed sinc FIR filter, order = 170 and transition band width = 10 Hz). Channels that had been identified as noisy in the ICA data copy were spherically spline‐interpolated in the filtered data. Data were segmented into epochs of 500 ms, including a 100‐ms prestimulus baseline, which was used for baseline correction. Epochs were removed from further analysis (*M* = 7.5% rejection rate) if signal range was larger than 100 μV at any channel. Epochs were averaged for each participant, condition and channel to obtain individual ERPs. Note, first‐deviant trials were collapsed irrespective of the stimulus that followed in Position 2. However, the stimuli in Position 2 were averaged separately for each stimulus (standard and deviant) and deviant repetition probability (high and low). The global standard included all standard‐after‐standard trials, except for the first five trials in each block to allow build‐up of the standard regularity, and the standard that followed a Position 2 stimulus.

#### 2.5.2 ICA Preprocessing

ICA was performed with the Adaptive Mixture Independent Component Analysis (AMICA) algorithm (Palmer et al. 2011). A copy of the original data was prepared by filtering the raw data with a 1‐Hz high‐pass and a 100‐Hz antialiasing low‐pass filter (Hamming windowed sinc FIR filters, high‐pass: order = 846, transition band width = 0.2 Hz; low‐pass: order = 170, transition band width = 10 Hz), and then downsampled to 256 Hz. Noisy channels (except ocular) were identified with a modified version of the *findNoisyChannels()* function from the PREP pipeline (Bigdely‐Shamlo et al. 2015). That is, they were removed if they either contained extreme amplitudes (robust channel deviation *z* score > 5), or an abnormally high electromagnetic noise level (*z* scored estimate of noise‐to‐signal ratio > 5; refers to ratio of power above 50 Hz to total signal power), or too low correlation with other channels (percentage of 1‐s windows with a correlation < 0.4 was above 1%). The data were epoched from −100 to 700 ms relative to stimulus onset, and no baseline correction was performed for ICA purposes. Epochs were removed if the voltage exceeded an absolute threshold of ± 500 μV, a delta threshold of 600 μV, or contained muscle activity defined as a deviation of +25 or −100 dB from baseline in the spectral range of 20–40 Hz. The latter criterion was chosen to remove eye blinks contaminated with muscle activity, to increase the probability of obtaining a clean blink IC.

### 2.6 Exploratory Factor Analysis (EFA)

We followed the tutorial by Scharf et al. (2022) on applying temporal exploratory factor analysis (EFA) to ERP data. Temporal EFA was applied to a selected time range (−100 to 400 ms relative to sound onset) of the within‐subject averages to estimate dependent measures of relevant components for inferential testing, as the temporal and spatial overlap of ERP components (such as N1 contributions in the MMN latency range) can bias estimates of amplitude, latency and topography. Temporal EFA aims to decompose the observed signal into a set of underlying factors that summarise sampling points with a similar activity pattern across participants, channels and conditions. The number of to‐be‐extracted factors was determined with the Empirical Kaiser Criterion. Unrotated factor loadings were estimated based on the covariance matrix, on which a Promax rotation (*κ* = 3) was applied to obtain the rotated solution. Factor loadings were standardised during rotation only, to avoid that large factors dominate the solution. Note that each factor is characterised by two coefficients: (a) *Factor loadings* reflect the time course of a factor (fixed across channels, conditions and participants), and (b) *factor scores* represent the contribution of each factor to the observed ERP wave (i.e., amplitude) for each observation. ERPs were reconstructed by obtaining the matrix product of the unstandardised loadings and the scores of each factor respectively (thus, the factor‐specific contribution of each factor is rescaled to the original unit μV) and then summing across these factor‐wise reconstructed ERPs.

### 2.7 Preprocessing of Behavioural Data

Response time (RT) was defined as the time between the onset of a tone and the key press attributed to it. A key press was attributed to a sound if it occurred 0–800 ms relative to tone onset. In accordance with signal detection theory (SDT), a button press attributed to a deviant sound (target) was treated as a hit, whereas a button press attributed to a standard sound following a first deviant (nontarget) was treated as a false alarm. For analysis of response times and false‐alarm rates, a Position 2 stimulus was excluded if the preceding deviant (Position 1) was missed. Hit rates were calculated by dividing the number of hits by the number of target trials for each deviant type (high and low repetition probability). False‐alarm rates were calculated as the number of key presses relative to the number of nontarget sounds (i.e., standards following a first deviant) per deviant type. As previous studies have demonstrated notable floor/ceiling effects in these accuracy measures, the decision criterion (*c*) was estimated in addition to quantify the response bias related for each repetition rule. Hit and false‐alarm rates were each corrected using the log‐linear rule (Hautus and Lee 2006) before applying an inverse normal transform and computing sensitivity index *d′* as their difference ($z_{\mathrm{hit}}^{*}-z_{\text{false alarm}}^{*}$) and the response criterion (bias) *c* as their sum multiplied by −0.5; $\left(-,0.5,\times ,\left(z_{\mathrm{hit}}^{*}+z_{\text{false alarm}}^{*}\right)\right)$. The median of all collected response times per condition (position and repetition probability) was used to aggregate the typically right‐skewed response time distribution on the participant level. Response measures of all first deviants (i.e., irrespective of actual repetition) were collapsed within high and low repetition probability, respectively. For the single‐trial analysis of response time data, each individual button press generated by a participant in response to a deviant sound was taken.

### 2.8 Statistical Analysis

Data analysis was run in R using the RStudio environment and a number of packages, mainly from the *tidyverse* (Wickham et al. 2019) such as *dplyr* (Wickham et al. 2021) and *tidyr* (Wickham and Henry 2020) for data wrangling, and *ggplot2* (Wickham 2016) to generate plots. Effect sizes were bootstrapped with *bootES* (Kirby and Gerlanc 2013). Bayesian analysis was implemented with *BayesFactor* (Morey and Rouder 2018) and the functions provided by van Doorn et al. (2020) for the nonparametric tests. Bayesian multilevel model analysis was implemented with *brms* (Bürkner 2017), *loo* (Vehtari et al. 2017) and *tidybayes* (Kay 2024). Bayes factors (*BF*_10_) were generally calculated as the ratio of evidence for the alternative hypothesis given the observed data, defined as a Cauchy prior distribution centred around 0 with a scaling factor of *r* = √2/2 (Rouder et al. 2009, 2012), and for the null hypothesis, which corresponded to a standardised effect size *δ* = 0. For the Bayesian equivalent of a *t* test, it reflects the likelihood of the alternative hypothesis (true effect is different from zero) relative to the null hypothesis (true effect is equal to zero) given the observed data. In the case of ANOVAs, *BF*_10_ reflects the likelihood of the model including the effect of interest relative to the null model given the observed data. This likelihood ratio can be easily inverted by taking the reciprocal (1/*BF*_10_) to express the likelihood of the null model relative to the model including the effect of interest (*BF*_01_). If not indicated otherwise, reported Bayes factors from ANOVAs reflect the comparison between a given model including an effect of interest and the null model including only a random intercept term for participants.

We followed the convention to categorise Bayes factors by magnitude (Jeffreys 1961; Lee and Wagenmakers 2014): A *BF*_10_ > 3 is taken as moderate evidence for the alternative hypothesis (*BF*_01_ < 0.3 for the null hypothesis); values closer to 1 are considered inconclusive or anecdotal evidence, respectively. Values larger than 10 are labelled as strong and values larger than 42 as very strong evidence for the alternative hypothesis (the respective reciprocal values as evidence for the null hypothesis). However, the Bayes factor is also directly interpretable as an odds ratio (Rouder et al. 2009).

#### 2.8.1 EFA Data

We implemented the same statistical analysis of the ERPs as in Coy et al. (2024), with one exception: Instead of analysing only one channel (FCz), here, we analyse the mean of a frontocentral region of interest (ROI: FCz, FC1, FC2 and Fz). The estimated peak amplitude of a given factor‐wise reconstructed ERP was used as the dependent variable in the statistical analyses. To assess whether the identified factors in the classic MMN and P3a latency range actually contributed to the (respective negative or positive) difference between deviants and standards (i.e., whether it represents MMN or P3a activity), Bayesian paired *t* tests were applied to the difference between first deviants (Position 1) and the global standard (corresponding to the traditional MMN analysis), for high and low deviant repetition probability, respectively, in each potential factor. Also, the deviant‐minus‐standard difference was compared between high and low deviant repetition probability. Subsequently, the thus verified factors were used to test for effects of deviant repetition probability on the Position 2 stimuli. Following MMN‐logic, the relevant difference was defined as rule violation minus rule confirmation: For deviants, this corresponds to the contrast low minus high, whereas for standards, it corresponds to high minus low repetition probability.

In addition, this logic was also used to assess both the effects of the conditional repetition rule associated with the respective preceding deviant (confirmation vs. violation based on deviant repetition probability) and the global rule (frequent standards vs. rare deviants) in one 2 × 2 Bayesian repeated‐measures ANOVA (*conditional rule* × *global rule*). This allows us to formally test whether the activity in response to a stimulus following a first deviant is best explained by either their (mis)match of the *conditional rule*, the *global rule* or a combination of both. To cover all possible combinations in one test statistic, an inclusion Bayes factor (*BF*_incl_) was computed for both main effects and the interaction effect. *BF*_incl_ expresses the probability of the observed data under all models containing a particular effect against that under all models without that effect (Hinne et al. 2020).

#### 2.8.2 Behavioural Data

To test whether the deviant repetition probability (high vs. low) affects target detection of the second relative to the first deviant, hit rates in deviant repetition trials (Position 2 stimulus is a deviant) were analysed by means of a 2 × 2 Bayesian repeated‐measures ANOVA, which included the within‐participant factors position (first vs. second deviant) and deviant repetition probability (high vs. low). The effect of deviant repetition probability (high vs. low) on false‐alarm rates (Position 2 standards) was investigated by means of a Bayesian equivalent of a nonparametric paired *t* test (van Doorn et al. 2020). A repeated‐measures 2 × 2 ANOVA compared the effect of position (first vs. second deviant) and deviant repetition probability (low vs. high) on response times for two successive deviants (deviant repetition). The *position* × *deviant repetition probability* interaction was deconstructed by a set of Bayesian paired *t* tests.

To investigate the time course of the facilitation effect related to the position of the deviant and the associated repetition rule over the course of the behavioural blocks, we fitted Bayesian multilevel models on single‐trial RT data, accounting for their hierarchical nature (i.e., single‐trial observations are nested in participants). As response time data are typically right‐skewed, a lognormal link‐function was used, which is defined by two parameters: *mu* (corresponds to the median RT) and *sigma* (higher sigma increases the mean but not the median RT). Note, both parameters are on the log‐scale. As can be seen in the overview of all fitted models given in Table 1, all fitted models contained a fixed slope for the position × repetition probability interaction for *sigma*, to account for differences in the variance within each cell. When the interaction term is included, this always implies that the respective main effects are also included in the model. The coefficients described in the following relate to the *mu* parameter. The null model only contained fixed slopes for the position × repetition probability interaction and a random intercept for participants (null model m0). We used a nonlinear function to estimate effects of time with the temporal predictor *trial #* corresponding to the absolute position of a given deviant trial within the behavioural part. The temporal effect on RTs was modelled as a power law (a * trial # ^*b*^) in two additional models (m1 and m2) that differed as to which fixed slopes were included in the estimation of the two constants (*a* and *b*). Specifically, m1 restricted the initial response time (coefficient *a*) to effects of position (first vs. second deviant), whereas in m2, this constant could also interact with deviant repetition probability (high vs. low). Informed priors (based on the previous studies using this paradigm) were placed on the fixed effect coefficients to enhance sampling efficiency in plausible parameter space; a detailed overview can be found in the analysis markdown document. For each model, we ran four chains with 6000 iterations each. Model convergence was established using potential scale reduction factors (Rhats < 1.01), effective sample size estimates (> 1000), visual inspection of trace plots and leave‐one‐out cross validation (LOO; Pareto *k* estimates < 0.7). Model comparison was based on the LOO‐information criterion (lower values indicating a better model fit) and the Bayes factor for a given unrestricted model against a restricted model. Post hoc hypotheses of relevant regression coefficients were tested by analysing the posterior distribution. We report the posterior mean of the relevant difference and 95% credible intervals (CI 95%) and the posterior probability (*PP*; percentage of posterior samples in favour of the specified hypothesis).

**TABLE 1:** Specification of Bayesian multilevel models for analysis of single‐trial RTs.

Model	Distribution parameter	Formula
m0	*mu*	RT ~ 1 + position * repetition probability + (1|participant)
	*sigma*	sigma ~ position * repetition probability
m1	*mu*	RT ~ *a* * *trial #* ^*b*^ *a* = 1 + position + (1|participant) *b* = 1 + position * repetition probability + (1|participant)
	*sigma*	sigma ~ position * repetition probability
m2	*mu*	RT ~ *a* * *trial #* ^*b*^ *a* = 1 + position * repetition probability + (1|participant) *b* = 1 + position * repetition probability + (1|participant)
	*sigma*	sigma ~ position * repetition probability

*Note:* The lognormal parameter estimates (*mu* and *sigma*) are on the log scale and translate to median and a dispersion parameter at the response scale. The intercept is represented by the digit 1, effects in brackets refer to random effects. If an interaction of two factors is included, the respective main effects are as well.

## 3 Results

### 3.1 EEG

**FIGURE 2:**
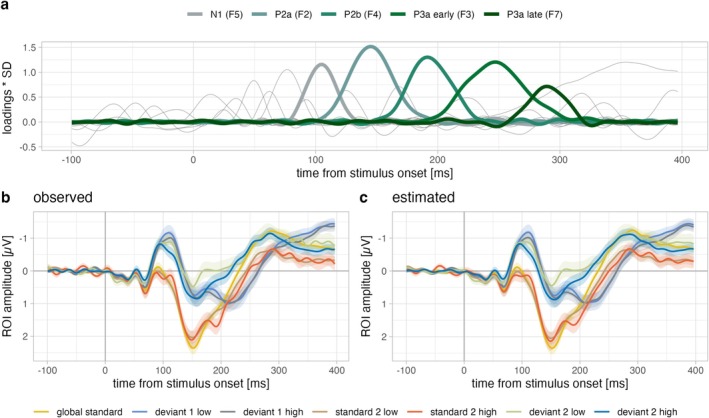
Grand‐averaged ERPs (*N* = 60) and the solution obtained with exploratory factor analysis. (a) Rotated unstandardised factor loadings (loading × SD) of the 23 retained factors. The factors chosen for further analysis (N1, P2a, P2b, P3a early and P3a late) are highlighted in shades of green. (b) Grand‐averaged observed ERPs at the frontocentral region of interest (ROI: FCz, FC1, FC2 and Fz) can be compared against (c) the corresponding reconstructed ERPs based on the obtained EFA solution (sum of all retained factors). Shaded ribbons represent ± 1 SEM.

The grand‐averaged ERPs are displayed in Figure 2 along with the results obtained from the temporal exploratory factor analysis (EFA). The unstandardised loadings of all 23 retained factors (as determined by Empirical Kaiser Criterion) are shown in Figure 2a. Grand‐average waves at the frontocentral ROI (Figure 2b, observed ERPs) visually correspond well with the EFA reconstruction waves (Figure 2c, estimated ERPs) of standard and deviant waves, suggesting that the EFA solution accurately represents the original data. Please note that factor numbers are ordered by explained variance, not by peak latency. The identification of factors of interest was based on polarity, latency and topographical information.

**FIGURE 3:**
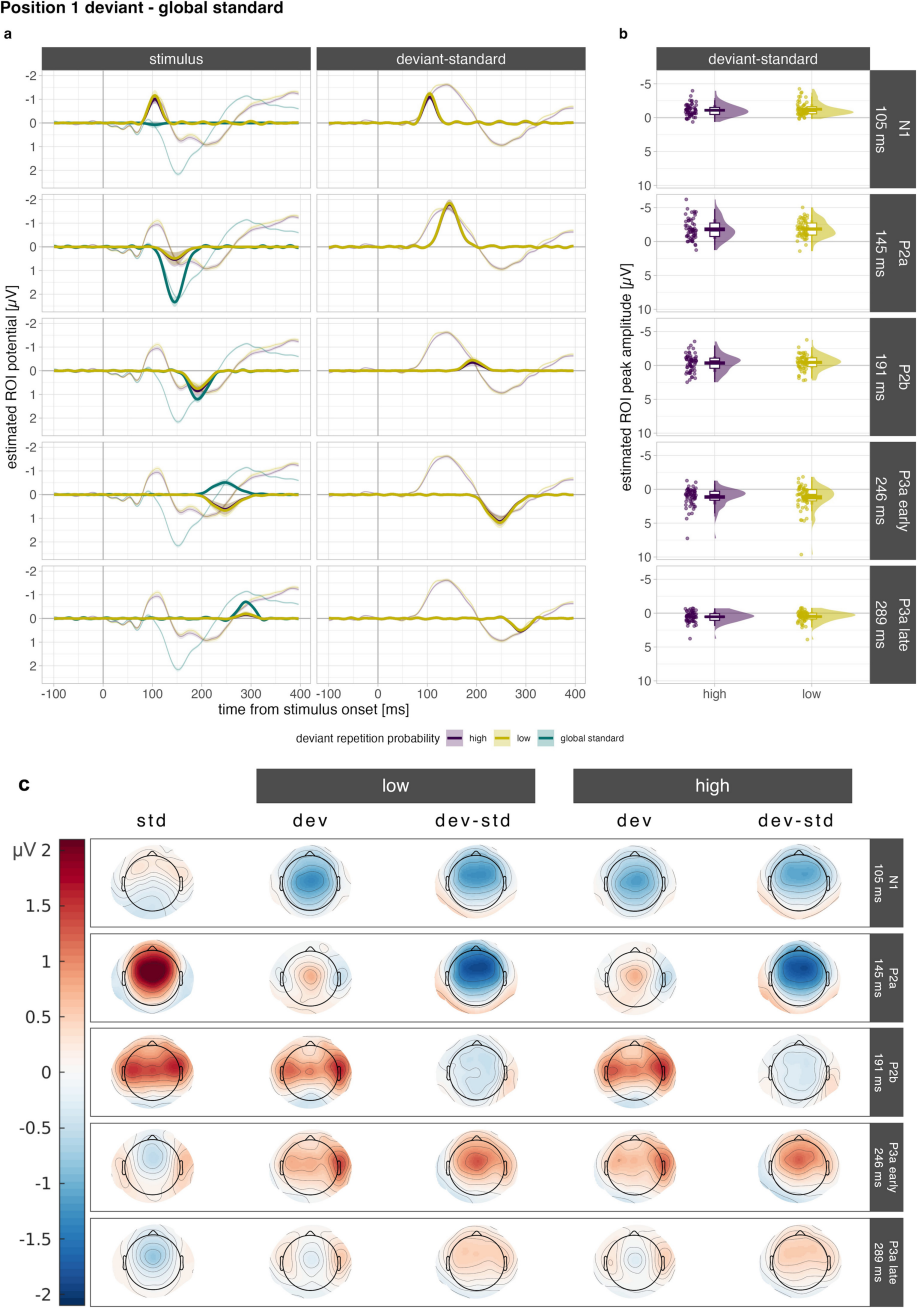
Reconstructed ERPs and the respective contribution of selected factors in Position 1 deviants of the frontocentral ROI. Factors are arranged from top to bottom and correspond to the following ERP components: Factor 5 (N1), Factor 2 (P2a), Factor 4 (P2b), Factor 3 (P3a early) and Factor 7 (P3a late). The reconstructed ERP waves are displayed in panel (a) both at the stimulus level (left) and for the respective deviant–standard difference (right). Reconstructed ERPs (sum of all retained factors) are depicted as thin lines, and the respective factor contribution is marked by the thicker lines. Shaded ribbons represent ± 1 SEM. The raincloud plots in panel (b) display the corresponding estimated peak amplitudes of the deviant–standard contrast for each deviant type. Each dot represents one observation (i.e., participant). Crossbars represent ± 1 SEM. (c) The topographies show average activity at the respective peak latency for each selected factor. The first column depicts the respective topography for the global standard (std), the second and the fourth columns for deviants (dev) and the third and fifth columns the respective deviant‐minus‐standard difference (dev‐std).

In the classical MMN time‐range (100–250 ms; Näätänen et al. 1978, 2007) there are three factors that all show a negative difference between deviants and standards with polarity inversion at the mastoids (see topographies in Figure 3c, Columns 3 and 5): Factor 5 peaking at 105 ms likely corresponds to N1 (more negative amplitudes for rare deviants relative to the frequent global standard) and two factors that, based on their positive polarity and latency, likely represent the P2 (less positive amplitudes for deviants relative to the global standard). Factor 2 peaking at 145 ms was identified as P2a (central topography at stimulus level) and Factor 4 peaking at 191 ms as P2b (bilateral topography at stimulus level). Factor 3 was identified as early P3a, because it captures the pronounced positive difference between deviants and global standards, peaking at 246 ms with amplitudes that are negative for standards and positive for deviants at the stimulus level. Factor 7 reflects the leg on the right‐hand side of the positive portion of the difference wave, peaking at 289 ms (less negative amplitudes for deviants) and was therefore identified as late P3a. Estimated peak amplitudes are reported in Table 2 for the five selected factors.

**TABLE 2:** Estimated peak amplitudes (μV) for each factor as a function of stimulus, position and deviant repetition probability in the frontocentral ROI.

Stimulus	Factor 5 (N1) 105 ms		Factor 2 (P2a) 145 ms		Factor 4 (P2b) 191 ms		Factor 3 (P3a_early_) 246 ms		Factor 7 (P3a_late_) 289 ms	
	*M*	(SE)	*M*	(SE)	*M*	(SE)	*M*	(SE)	*M*	(SE)
Global standard	0.078	(0.148)	2.336	(0.197)	1.207	(0.144)	−0.513	(0.135)	−0.704	(0.067)
Position 1										
Deviant high	−1.013	(0.197)	0.535	(0.301)	0.855	(0.218)	0.624	(0.222)	−0.167	(0.108)
Deviant low	−1.163	(0.226)	0.491	(0.273)	0.761	(0.219)	0.672	(0.272)	−0.195	(0.117)
Deviant—global standard										
High	−1.091	(0.112)	−1.802	(0.195)	−0.352	(0.163)	1.137	(0.178)	0.537	(0.100)
Low	−1.242	(0.127)	−1.845	(0.158)	−0.446	(0.146)	1.185	(0.232)	0.508	(0.104)
Position 2										
Deviant high	−0.755	(0.167)	0.784	(0.258)	0.352	(0.200)	−0.681	(0.182)	−0.599	(0.101)
Deviant low	−0.902	(0.221)	0.413	(0.253)	0.008	(0.246)	−0.579	(0.258)	−0.569	(0.158)
Deviant: Low–high	−0.146	(0.207)	−0.371	(0.228)	−0.343	(0.219)	0.101	(0.217)	0.031	(0.136)
Standard high	0.059	(0.195)	2.147	(0.295)	1.565	(0.237)	0.082	(0.194)	−0.489	(0.126)
Standard low	0.143	(0.157)	2.078	(0.196)	1.255	(0.166)	−0.147	(0.164)	−0.403	(0.100)
Standard: High–low	−0.084	(0.171)	0.068	(0.200)	0.310	(0.203)	0.229	(0.163)	−0.086	(0.114)

#### 3.1.1 Position 1 Deviants Versus Global Standard (Sanity Check)

In Figure 3a, the contribution of each selected factor to the whole estimated ERP (sum of all factors) in the ROI is displayed for first deviants (Position 1: high and low) and the global standard, as well as their respective difference (Figure 3b). As can also be seen in the topographies provided in Figure 3c, the deviant‐standard difference is characterised by a clear frontocentrally distributed negative deflection, with polarity inversion at the mastoids, peaking around 150 ms (MMN latency range), a positive deflection peaking around 250 ms (P3a latency range) and a later negative‐going potential. The distribution of the estimated peak amplitude values across participants for the deviant‐minus‐standard difference is displayed in Figure 3b.

There is very strong evidence that N1 (Factor 5) is more negative for first deviants compared to the global standard both when repetition probability is high (*BF*_10_ = 1033 × 10^8^, *d*_z_ = −1.26) and low (*BF*_10_ = 1281 × 10^8^, *d*_z_ = −1.26), in both cases mainly contributing to the early portion of the negative difference in the MMN range. Here, the estimated peak amplitude is more negative when repetition probability is low than when it is high (descriptively slightly larger N1 for the deviant with the lower base rate), but the evidence is moderately in favour of the null hypothesis (*BF*_10_ = 0.233, *d*_z_ = −0.162).

P2a (factor 2) peaks at 145 ms and shows a clear negative difference between first deviants and the global standard, covering the largest portion of the total negative deflection of the deviant‐standard difference within the MMN latency range. There is very strong evidence for this difference being negative, for both high (*BF*_10_ = 1661 × 10^7^, *d*_z_ = −1.19) and low (*BF*_10_ = 1004 × 10^11^, *d*_z_ = −1.51) repetition‐probability first deviants. Again, the null hypothesis is moderately favoured in the high versus low contrast of this difference (*BF*_10_ = 0.144, *d*_z_ = −0.03). The slightly later P2b (factor 4; peak at 191 ms) also exhibits a negative difference for both high (*BF*_10_ = 1.219, *d*_z_ = −0.28) and low (*BF*_10_ = 9.144 *d*_z_ = −0.40) repetition‐probability first deviants, but of smaller effect size. Again, the null hypothesis is moderately favoured in the high versus low contrast of this difference (*BF*_10_ = 0.178, *d*_z_ = −0.08). This indicates that the contribution at the level of P2 (Factors 2 and 4) to the observed MMN (i.e., the observed negativity in the MMN latency range) is of similar magnitude in response to a first deviant irrespective of whether it is associated with high or low repetition probability.

As can be seen in Figure 3a, at the level of the global standard, Factors 3 and 7 correspond to negative deflections, peaking at 246 and 289 ms, respectively. Bayesian *t* tests of the ROI peak show very strong evidence that deviant ERPs are more positive than global standard ERPs both when their repetition probability is high (P3a early: *BF*_10_ = 4674 × 10^2^, *d*_z_ = 0.83; P3a late: *BF*_10_ = 10,370, *d*_z_ = 0.69) and low (P3a early: *BF*_10_ = 4756, *d*_z_ = 0.66; P3a late: *BF*_10_ = 2297, *d*_z_ = 0.63). Latency and polarity of this difference between deviants and standards indicate that Factor 3 corresponds to the observed early P3a and Factor 7 to a late P3a. The null hypothesis is moderately favoured in the comparison of high and low repetition probability of the deviant‐standard difference in early P3a (*BF*_10_ = 0.147, *d*_z_ = 0.03) and late P3a (*BF*_10_ = 0.148, *d*_z_ = −0.04), indicating that in first deviants early and late P3a are of similar magnitude regardless of the repetition probability associated with the deviant.

#### 3.1.2 Position 2 Stimuli

**FIGURE 4:**
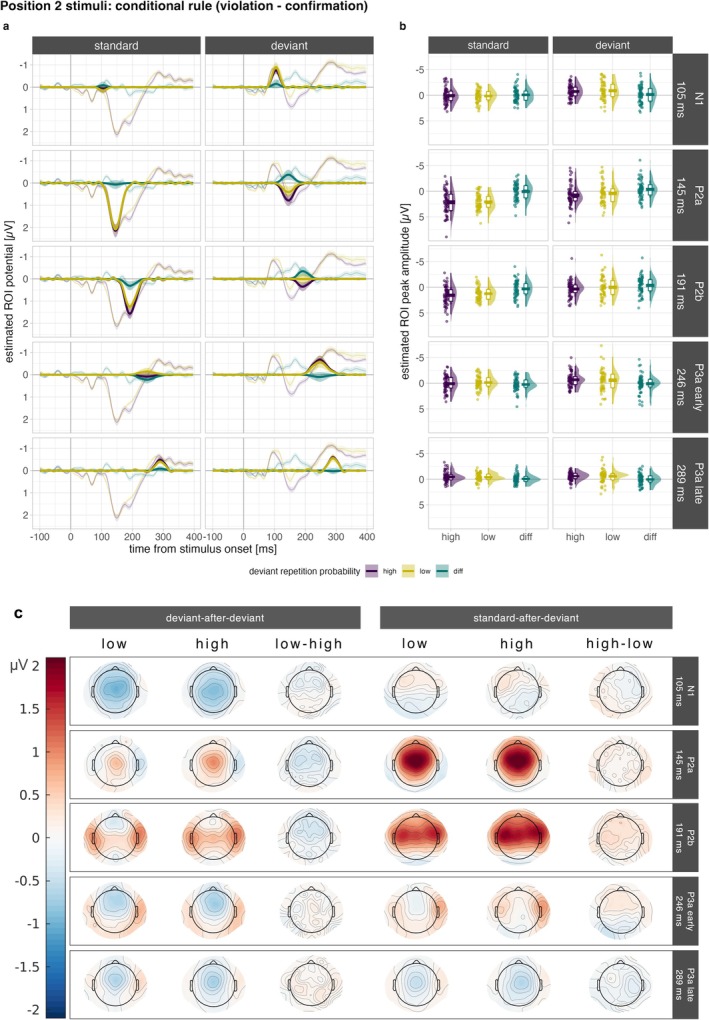
Reconstructed ERPs and the respective contribution of selected factors in Position 2 stimuli in the frontocentral ROI. Factors are arranged from top to bottom and correspond to the following ERP components: Factor 5 (N1), Factor 2 (P2a), Factor 4 (P2b), Factor 3 (P3a early) and Factor 7 (P3a late). Reconstructed ERPs (sum of all retained factors) are depicted in thin lines; the respective factor contribution is marked by the thicker lines. The left side of panel (a) displays standard‐after‐standard, and the right‐side deviant‐after‐deviant reconstructed ERP waves for the following conditions: high repetition probability (purple), low repetition probability (yellow) and respective rule‐violation minus rule‐confirmation difference (green). This refers to the high–low contrast for Position 2 standards and to low–high contrast for Position 2 deviants. Shaded ribbons represent ± 1 SEM. The raincloud plots in panel (b) display the corresponding estimated peak amplitude values of this contrast for each stimulus and the relevant difference. Each dot represents one observation (i.e., participant). Crossbars represent ± 1 SEM. (c) The topographies show average activity at the respective peak latency for each selected factor.

In Figure 4a, the contribution of each selected factor to the whole estimated ERP (sum of all factors) in the ROI is displayed for Position 2 stimuli, that is, deviants and standards following a first deviant, which are contrasted by deviant repetition probability. The distribution of the estimated peak amplitude values across participants is displayed on the stimulus level with the respective difference (deviant: low‐high; standard: high‐low) in Figure 4b. Corresponding topographies are depicted in Figure 4c.

##### 3.1.2.1 Standard After Deviant

There is moderate evidence against an effect of deviant repetition probability on estimated peak amplitudes of factors within the MMN time range for components N1 (Factor 5: *BF*_10_ = 0.159, *d*_z_ = −0.06) and P2a (Factor 2: *BF*_10_ = 0.149, *d*_z_ = 0.04), but evidence is inconclusive for P2b (Factor 4: *BF*_10_ = 0.426, *d*_z_ = 0.20). When a first deviant is directly followed by a standard, there is inconclusive to moderate evidence against P3a elicitation (Factors 3 and 7) in response to that standard when the preceding deviant is associated with a high probability of repetition (rule violation) compared to a deviant associated with a low (rule confirmation) probability of repetition (P3a early: *BF*_10_ = 0.358, *d*_z_ = 0.18; P3a late: *BF*_10_ = 0.186, *d*_z_ = −0.10). Thus, evidence is not in favour of MMN or P3a activity in response to rule‐violating compared to rule‐conforming standards that follow a first deviant.

##### 3.1.2.2 Deviant After Deviant

Descriptively, within the MMN time window, the average estimated peak amplitude of a deviant is more negative when the preceding deviant is associated with a low probability of repetition (nonrepetition rule) compared to a high probability of repetition (repetition rule). However, at the level of N1, there is moderate evidence against an effect (Factor 5: *BF*_10_ = 0.179, *d*_z_ = −0.09), and at the level of P2a and P2b, evidence is inconclusive (Factor 2: *BF*_10_ = 0.493, *d*_z_ = −0.21; Factor 4: *BF*_10_ = 0.448, *d*_z_ = −0.20). Also, there is no indication of early or late P3a elicitation; that is, there is moderate evidence for the null hypothesis for low‐ compared to high‐repetition‐probability second deviants (Factor 3: *BF*_10_ = 0.157, *d*_z_ = 0.06; Factor 7: *BF*_10_ = 0.145, *d*_z_ = 0.03).

##### 3.1.2.3 Conditional Versus Global Rule

There is very strong evidence for the inclusion of the main effect of the *global rule* (rare deviants vs. frequent standards) to explain the variance in Position 2 stimuli in all factors within the MMN time range (Factor 5 [N1]: *BF*_incl_ = 2184 × 10^5^; Factor 2 [P2a]: *BF*_incl_ = 4174 × 10^12^; Factor 4 [P2b]: *BF*_incl_ = 5558 × 10^7^). That is, on average, N1 is larger, and P2a and P2b are less positive in response to deviants than in response to standards (all yielding a negative deviant–standard difference), indicating the elicitation of MMN in response to a violation relative to a confirmation of the global rule. On average, at the level of N1, P2a and P2b, there is moderate evidence against the inclusion of the main effect of the *conditional rule* (Factor 5: *BF*_incl_ = 0.199; Factor 2: *BF*_incl_ = 0.227; Factor 4: *BF*_incl_ = 0.141). Regarding the *global rule* × *conditional rule* interaction term, there is moderate evidence against inclusion for N1 (Factor 5: *BF*_incl_ = 0.202), but evidence is inconclusive for P2a (Factor 2: *BF*_incl_ = 0.482) and P2b (Factor 4: *BF*_incl_ = 1.507). In the P3a time window, there is strong evidence for a deviant‐standard difference in the estimated peak amplitude (inclusion of main effect of *global rule* in Factor 3: *BF*_10_ = 442). Surprisingly, early P3a peak amplitudes are more negative for deviant after deviants than standard after deviants. There is inconclusive evidence regarding the inclusion for late P3a (main effect of *global rule* in Factor 7: *BF*_10_ = 0.348). There is moderate evidence against the inclusion of the main effect of *conditional rule* for both early and late P3a (factor 3: *BF*_incl_ = 0.264; factor 7: *BF*_incl_ = 0.146). Regarding the *global rule* × *conditional rule* interaction, there is moderate evidence against inclusion for early P3a (Factor 3: *BF*_incl_ = 0.218) and for late P3a (Factor 7: *BF*_incl_ = 0.228). Overall, when a stimulus follows a first deviant, there is strong evidence of large effects in the MMN time range (N1: *d*_z_ = 0.74; P2a: *d*_z_ = 0.85; P2b: *d*_z_ = 0.85) and small to medium‐sized effects in the P3a time range (early P3a: *d*_z_ = 0.43; late P3a: *d*_z_ = 0.17) when that stimulus violates the global rule (deviant) compared to when it conforms to the global rule (standard). Compared to the medium‐to‐large effect sizes of the global rule, the effects of the conditional rule are negligible.

### 3.2 Behaviour

#### 3.2.1 Accuracy

##### 3.2.1.1 Hits

**FIGURE 5:**
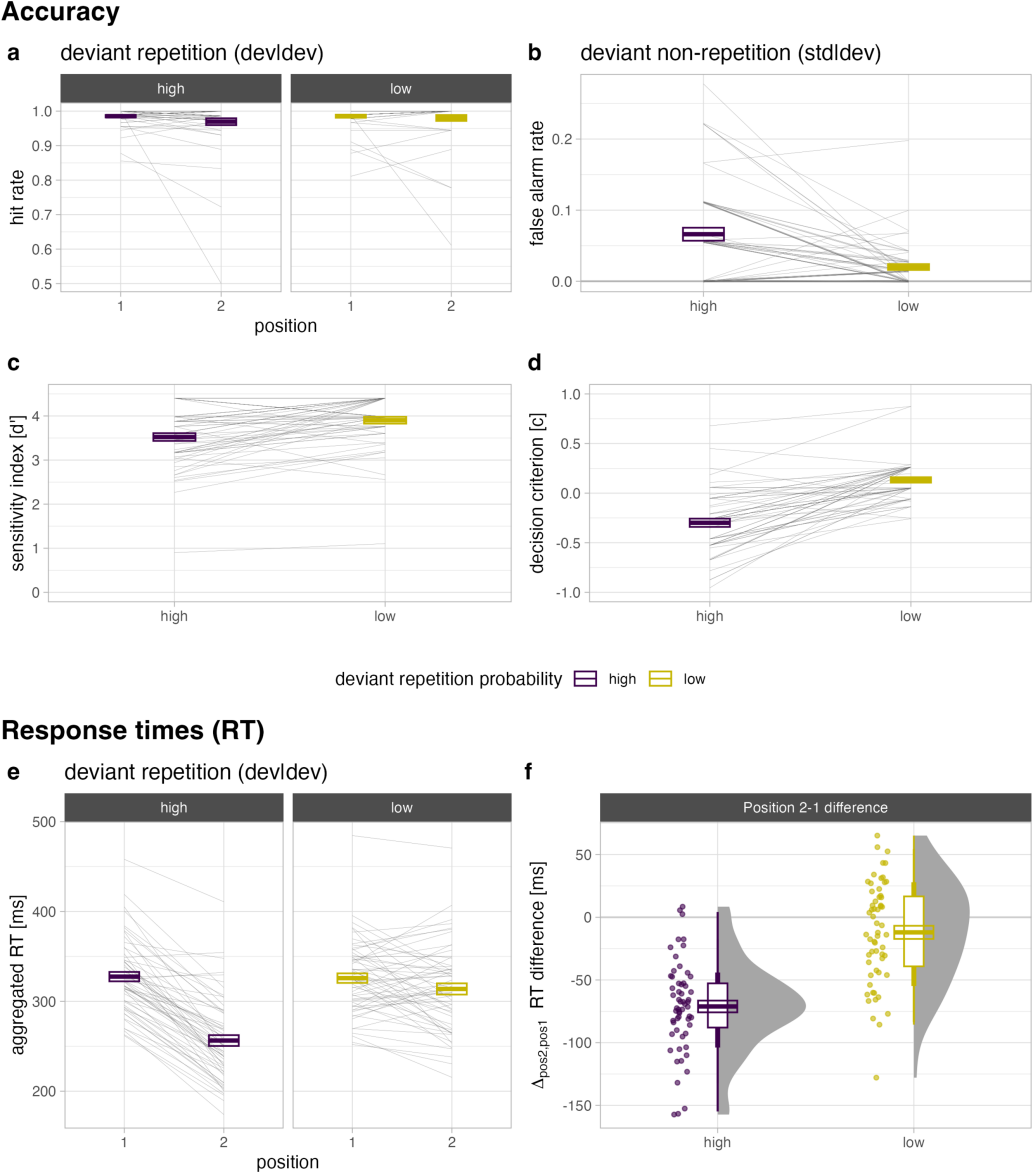
Behavioural data from the active deviant detection task (*N* = 59). (a) Hit rates when deviants were repeated as a function of position (first vs. second deviant) and deviant repetition probability (high vs. low) at the stimulus level. (b) False‐alarm rates in response to standard after deviants as a function of deviant repetition probability. (c) The ability to discriminate between deviants (targets) and standards (nontargets) and (d) the response bias (i.e., negative values indicate a bias towards responding with a button press; positive values indicate a tendency to withhold the button press) is compared between high and low deviant repetition probability. Median response times (in ms) are shown (e) at the stimulus level as a function of position and deviant repetition probability and (f) and the respective difference between first and second deviant (Position 2–1). Each observation (i.e., participant) is either represented by the thin grey lines (spaghetti plots) or the coloured dots (raincloud plots). Crossbars represent ± 1 SEM.

In the active deviant detection task, participants correctly responded to most targets; that is, hit rates are overall high (> 96% in all cells; Figure 5a and Table 3). The Bayesian repeated‐measures ANOVA yielded inconclusive evidence regarding the inclusion of a main effect of position (*BF*_10_ = 1.032), moderate evidence against including the main effect of repetition probability (*BF*_10_ = 0.239) or an additive model (*position + repetition probability*: *BF*_10_ = 0.247) and strong evidence against the inclusion of the interaction term (*position* × *repetition probability*: *BF*_10_ = 0.075).

**TABLE 3:** Behavioural data.

Deviant repetition probability		Position 1		Position 2		*Δ*_21_	
		*M*	(SE)	*M*	(SE)	*M*	(SE)
Accuracy							
Hit rate	High	0.985	(0.004)	0.969	(0.010)	−0.016	(0.009)
	Low	0.985	(0.004)	0.980	(0.009)	−0.005	(0.007)
False‐alarm rate	High			0.066	(0.009)		
	Low			0.020	(0.004)		
Sensitivity index (*d*′)	High			3.522	(0.088)		
	Low			3.904	(0.077)		
Decision criterion (*c*)	High			−0.299	(0.042)		
	Low			0.133	(0.027)		
Response times (ms)							
Deviant repetition	High	328	(5.2)	256	(6.0)	−71	(4.7)
	Low	326	(5.3)	314	(6.2)	−12	(5.2)

##### 3.2.1.2 False Alarms

As can be seen in Figure 5b and Table 3, key presses also occur in response to standards that directly follow a single deviant—that is, participants produced false alarms. The rate of false alarms increases on average by 4.6 percentage points (SE = 0.9) when deviant repetition probability is high compared to low. The evidence for this effect can be considered as very strong (*BF*_10_ = 2097, *d*_z_ = −0.85).

##### 3.2.1.3 Sensitivity Index (*d*′)

There is very strong evidence (*BF*_10_ = 2410) that on average participants discriminate worse (Figure 5c) between deviant (target) and standard (nontarget) sounds following a first deviant when deviant repetition probability is high (*M* = 3.522; SE = 0.088) than when it is low (*M* = 3.904; SE = 0.077).

##### 3.2.1.4 Response Bias (*c*)

There is very strong evidence (*BF*_10_ = 7259 × 10^8^) that on average participants use a more liberal response criterion (i.e., increased tendency to respond with a button press; Figure 5d) when the preceding deviant is associated with a high (*M* = −0.299; SE = 0.042) compared to low (*M* = 0.133; SE = 0.027) probability of repetition.

#### 3.2.2 Response Times

As can be seen in Figure 5e,f, almost all responses (except three participants with a positive RT difference) become faster from first to second deviant when repetition probability is high but not when it is low (Table 2). There is very strong evidence that the model including the *position × repetition probability* interaction (*BF*_10_ = 7608 × 10^31^) is to be preferred over the additive model (*BF*_10_ = 1090 × 10^19^) of both factors, *BF*_10_ = 6980 × 10^9^. There is very strong evidence that median response times decrease from first to second deviant (*M* = −71 ms; SE = 4.7 ms) when repetition probability is high, *BF*_10_ = 5755 × 10^15^, *d*_z_ = −1.97. When repetition probability is low, median response times on average decrease by 12 ms (SE = 5.2 ms) from first to second deviant (i.e., responses become faster), but there is only anecdotal evidence for the alternative over the null hypothesis, *BF*_10_ = 1.598, *d*_z_ = −0.30.

##### 3.2.2.1 Time‐Course

**FIGURE 6:**
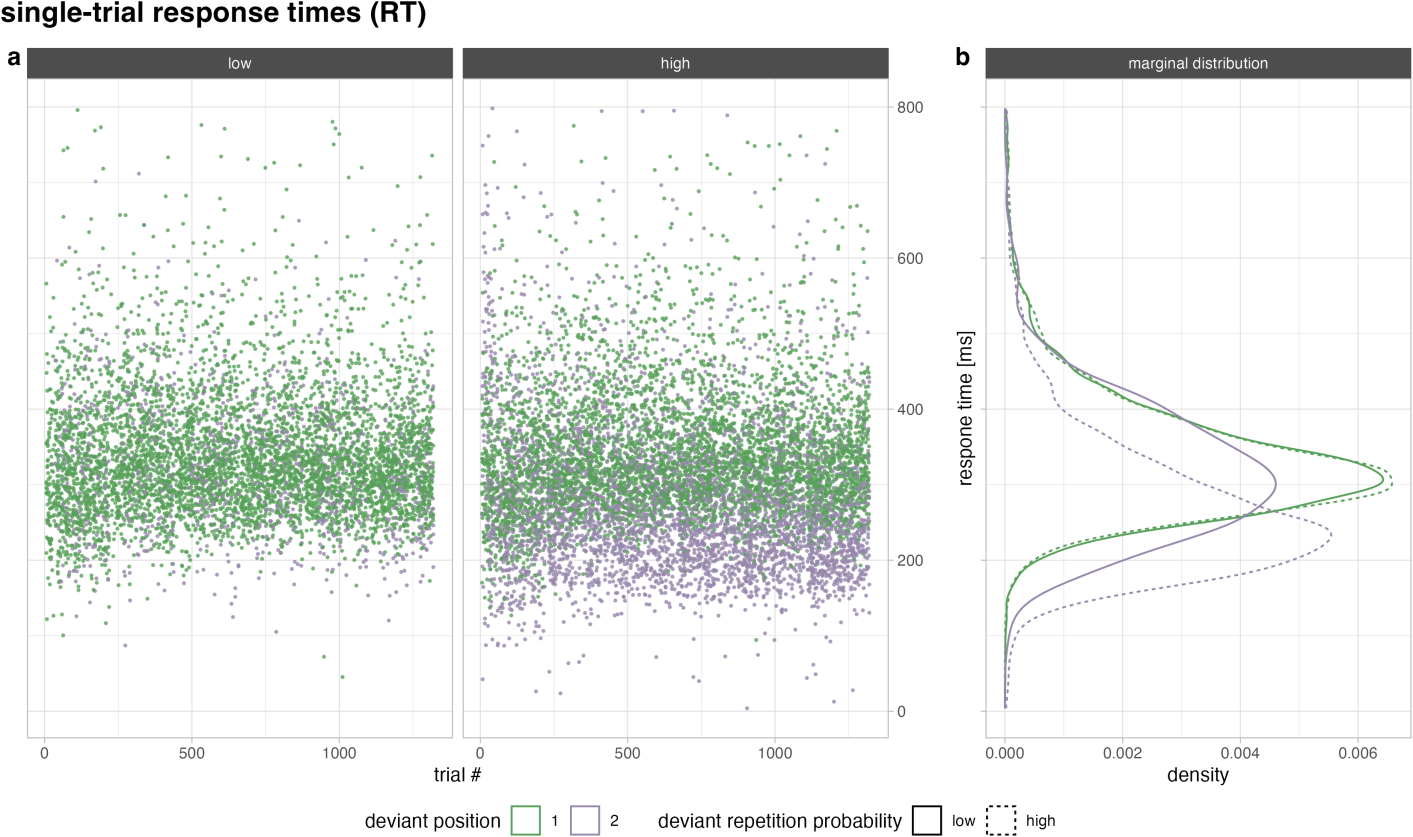
Observed single‐trial response times to deviants. RTs (a) as a function of time (trial #) and (b) their marginal distribution. The position of the deviant is represented through the colour (first deviant in green; second deviant in purple) and the repetition probability by the line type (low as solid; high as dashed). Each dot represents the response in a single trial by a participant. Note that the *y* axis is shared between the left and right plot.

Figure 6 depicts the distribution of all single‐trial response times as a function of deviant position (first or second) and deviant repetition probability (high vs. low). Whereas the distribution of first‐deviant responses is more or less congruent between high and low repetition probability (green lines), response times are more dispersed for second deviants (purple lines). Second deviants show a stronger skew to the left when associated with a high (dashed purple) than with a low (solid purple) probability of repetition.

The model containing the trial number of the deviant as a nonlinear temporal predictor (*LOOIC* = 176,111) is to be preferred over the null model only including fixed slopes for interaction and main effects of position and deviant repetition probability (*LOOIC* = 176,606); m1 vs. m0: *BF*_10_ = 4753 × 10^66^; Δ*LOOIC* = −495.27 (*SE*_ΔLOOIC_ = 53.33). This model only allows initial response times to vary by position (first vs. second deviants); Model 2 additionally includes the position × deviant repetition probability interaction effect (*LOOIC* = 176,115). However, there is very strong evidence that this does not improve model fit (m2 vs. m1: *BF*_10_ = 0.022), which is also indicated by a higher LOOIC (m2 vs. m1: Δ*LOOIC* = −3.93; *SE*_ΔLOOIC_ = 1.88).

**FIGURE 7:**
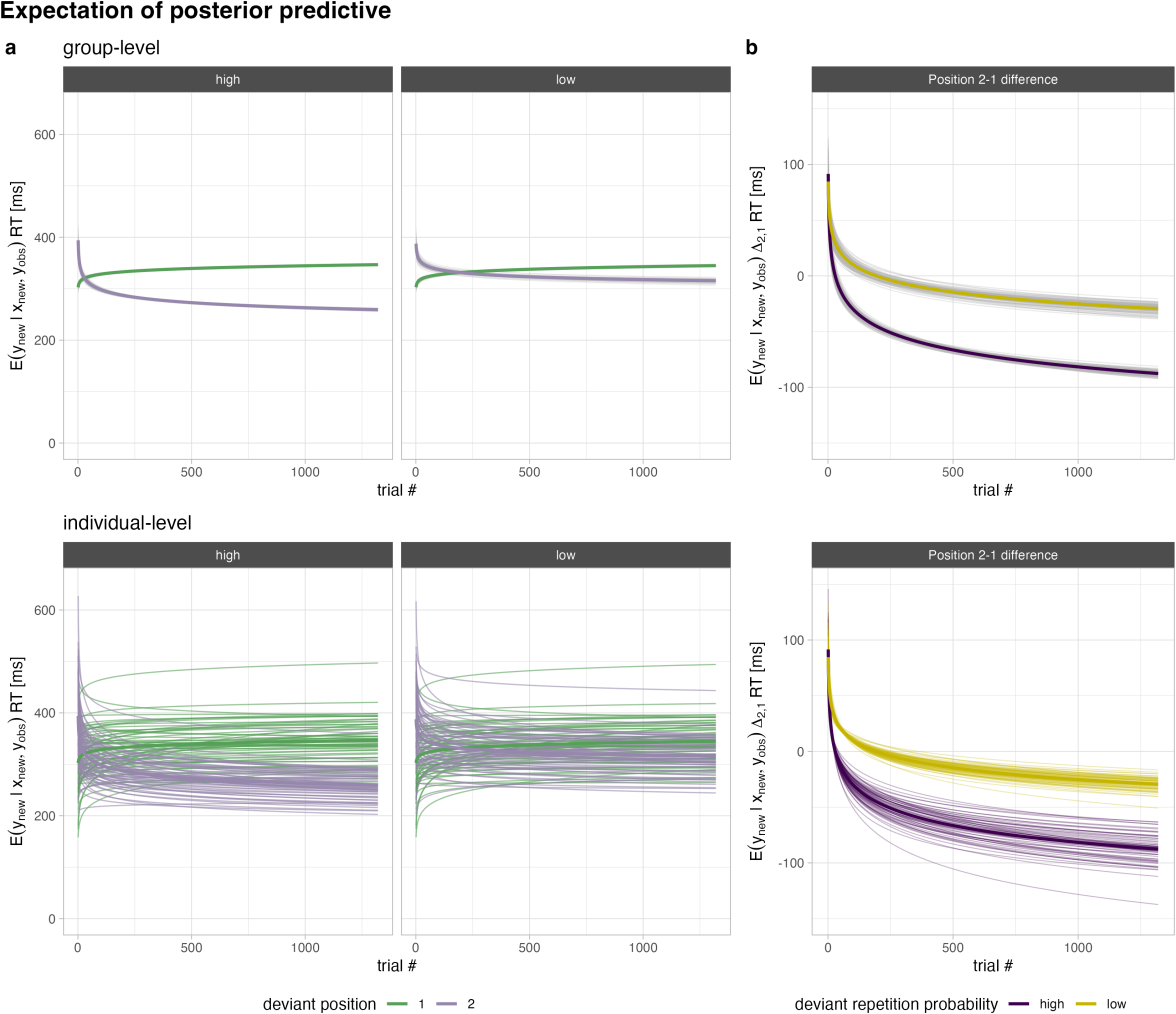
Expectation of posterior predictive of model m1. Predictions were generated over the whole parameter space of the temporal predictor (trial #) for each cell (position × deviant repetition probability) for 59 participants, based on 100 draws from the posterior. In the left panel, estimates are displayed for each condition (deviant position and repetition probability); in the right panel, it is the estimate of the difference between second and first deviant. Bottom: Here, the expectation of the posterior predictive reflects the mean expected value of the prediction for each participant (i.e., each line represents the individual time course of the median RT) across all draws. Top: Here, the point estimate is aggregated across all participants (group level: average of time courses of the median). The thin grey lines represent the point estimate for each draw from the posterior and the coloured thick lines the respective average of all draws, reflecting the variability of the parameter estimates between draws.

Figure 7 depicts the expectation of the posterior predictive for the preferred model (m1), that is, the expected value at the group level (top row) and at the participant level (bottom row). A comparison of the posterior predictive against the observed data (Figure S1) can be found in Part A of the Supporting Information, and some further information (parameter overview, posterior predictive check) on the model fit is provided in the analysis markdown on OSF. The following post hoc tests also refer to the preferred model (m1). In the beginning of the behavioural part, the expected median response time at group level is approximately 78 ms slower for a second relative to a first deviant (see Table 4 for detailed coefficient summary). As can be seen in the left part of Figure 7 (purple lines) and in Table 4, median response times decrease for second deviants with time (main effect of *position* on exponent *b*) but stronger when repetition probability is high compared to low (Table 4 exponent *b*: *position* × *deviant repetition probability* interaction; slope of deviant‐after‐deviants with high repetition probability: *b* = −0.01360, *PP* ≥ 0.999, *evidence ratio* ≥ 12,000). Response times for first deviants actually increase over time (Figure 7 green lines; Table 4 intercept of exponent *b*) but more so for first deviants that are typically followed by a second deviant (high repetition probability) than for first deviants typically followed by a standard (low repetition probability; Table 4: main effect of *deviant repetition probability* on exponent *b*; slope of first deviants with high repetition probability: *b* = 0.03812, *PP* ≥ 0.999, *evidence ratio* ≥ 12,000). In the rightmost column of Figure 7, it can be seen that the facilitation effect from first to second deviant is conditional on time. This implies that the marginal facilitation effect increases with the duration of the experiment.

**TABLE 4:** Estimates of fixed effects in the response time model including the temporal predictor (m1).

Coefficient	Parameter	Posterior estimates (log‐scale)		CI 95%	Hypothesis	PP	Evidence ratio
		*M*	(Error)				
*a*	Intercept	5.66933	(0.03238)	[5.61605; 5.72254]			
	Position 2	0.23541	(0.03325)	[0.18041; 0.28978]	> 0	≥ 0.9999	≥ 12,000
*b*	Intercept	0.00367	(0.00077)	[0.00243; 0.00496]	> 0	≥ 0.9999	≥ 12,000
	Position 2	−0.00818	(0.00092)	[−0.00968; −0.00665]	< 0	≥ 0.9999	≥ 12,000
	Repetition Probability high	0.00014	(0.00011)	[−0.00003; 0.00031]	> 0	0.9064	9.69
	Position 2 × repetition Probability high	−0.00542	(0.00028)	[−0.00588; −0.00495]	< 0	≥ 0.9999	≥ 12,000

*Note:* The intercept relates to the first deviant associated with low probability of repetition (reference category). The posterior estimates of the fixed slope parameters represent the difference of a given coefficient between the selected cell and the reference category. *PP* refers to the percentage of posterior samples in favour of the specified hypothesis. The evidence ratio expresses the percentage of posterior samples in favour of that specified hypothesis and the percentage of posterior samples against that hypothesis. All parameter estimates are on the log scale.

## 4 Discussion

The human auditory system readily extracts regularities of varying complexity from previously encountered sound sequences (Näätänen et al. 2007; Paavilainen 2013; Winkler 2007). It is believed that the rules governing the relationship of the sounds (i.e., the regularities) are used to form a prediction about what sound is likely to appear in the imminent future (Fitzgerald and Todd 2020; Garrido et al. 2009; Näätänen et al. 2001). However, a crucial factor has not received much explicit consideration so far: In the experiments underlying the aforementioned findings, the rule in question is typically encountered with a high frequency (Schröger et al. 2023). Thus, it is not sufficiently clear yet whether rarely encountered rules can also inform auditory prediction.

In a modified version of the classic oddball paradigm (Coy et al. 2022), we consistently associated two deviants of different pitch with diametrically opposed repetition rules within the same stimulation sequence: One deviant mostly occurred in pairs (high probability of repetition), whereas the other deviant mostly occurred in isolation (low probability of repetition). This design dissociates potential effects of conditional stimulus probability (high vs. low probability of deviant repetition) from effects of global stimulus probability (rare deviants vs. frequent standards). At the behavioural level, we successfully replicate earlier findings (Coy et al. 2022, 2024), demonstrating that conditional probability is extracted when deviants are task relevant. The findings at the level of event‐related‐potentials (ERPs) indicate that global probability rather than conditional probability is reflected in MMN and P3a components during passive listening (i.e., when all sounds are task irrelevant).

### 4.1 Behavioural Indicators of Deviant Repetition Rule Extraction

We successfully replicated previous behavioural findings (Coy et al. 2022, 2024) in the control task after the passive‐listening task (EEG part). Participants were asked to actively detect deviants but were not told about the deviant repetition rules. The false‐alarm rate was higher in response to standards that followed a deviant associated with high (unpredictable nonrepetition) than with low (predictable nonrepetition) repetition probability. Discrimination performance (sensitivity index *d*′) between deviants (targets) and standards (nontargets) following a first deviant (i.e., in Position 2) were worse when repetition probability was high compared to low. This effect is mainly driven by the corresponding higher rate of false alarms. On average, participants used a more liberal decision criterion when the conditional probability of another deviant (target) was high compared to low. Median response times decreased from first to second deviant when repetition probability was high (predictable deviant repetition), but there was no such facilitation when repetition probability was low (unpredictable deviant repetition). In an additional analysis, we investigated whether the response time facilitation effect is conditional on time of exposure. Indeed, our data suggest that in the beginning of the behavioural part, response times are generally slower for second relative to first deviants but do not differ between deviant repetition rules. We observed three temporal trends following a power law in our data: First, median response times for first deviants increase slightly with time. Second, response times to second deviants generally decrease with time. Third, the facilitation of second deviants over time is conditional on their repetition probability. This emerges as a larger and steeper decrease of response times for second deviants when their occurrence is conditionally likely (high repetition probability) compared to unlikely (low repetition probability). The first temporal trend might be related to some form of time penalty related to evaluating a first deviant with respect to whether a response is likely to be required for the subsequent stimulus, although it could also be related to fatigue. The second temporal trend shows that participants generally get faster over time with responding to second deviants, even when they are conditionally unlikely. This might be due to a reduction of the surprise over time about the imminent occurrence of a second deviant, or a general improvement at executing two responses shortly after each other. Importantly, the third temporal trend indicates that the facilitation effect emerges considerably faster and progresses towards a higher asymptote for conditionally likely deviant repetitions.

When considering that processing is facilitated when targets are predictable (Los and Schut 2008), this result pattern indicates that although participants had already been exposed to the sound sequences for approximately 1.5 h, the repetition rules associated with the deviants did not instantly drive behaviour, but only after some time spent on the task. One explanation could be that the translation of existing auditory predictions into action requires some familiarisation with the task in general. Another explanation could be that the rules associated with the rare deviants were never extracted during passive listening, and hence, they had to be newly acquired during the active listening task (also taking some time to emerge). These findings highlight the value of accounting for temporal trends in empirical data (Widmann et al. 2025), as the length of the experimental task might determine the magnitude of the marginal effect (e.g., in this design, the temporal trend indicates that the longer the behavioural part, the larger the observed marginal response time facilitation will be, until some asymptote is reached). This would explain why the facilitation effect from first to second deviant when the repetition is likely (high deviant repetition probability) was numerically largest in the first study utilising this paradigm (Coy et al. 2022); it had 12 instead of six blocks of the active task (with the same number of stimuli per block in both studies). In any case, the differential effects on performance already observed in the two earlier studies (Coy et al. 2022, 2024) and now replicated in the current one provide evidence that the predictive information carried by deviants is extracted and used to infer predictions when deviants are explicitly task relevant.

### 4.2 EEG Decomposition With EFA

As expected based on the vast literature on auditory regularity violation processing, the event‐related potential (ERP) in response to first deviants compared to the global standard is characterised by a pronounced frontocentral negativity peaking around 150 ms after sound onset, which was identified as mismatch negativity (MMN) and a frontocentral positivity peaking around 250 ms identified as P3a. Exploratory factor analysis (EFA) revealed that the negative deviant‐minus‐standard difference in the MMN time range receives spatially and temporally overlapping contributions from ERP components identified as N1 and P2. Interestingly, the P2 consisted of two subcomponents, which we labelled P2a (peak at 145 ms) and P2b (peak at 191 ms) in accordance with a recent suggestion (Steinmetzger and Rupp 2024). At the stimulus level, the earlier P2a is characterised by a frontocentrally distributed positivity, whereas the later P2b shows a bilateral more central positive distribution. A reinspection of the EFA solution obtained in our preceding study (Coy et al. 2024; EFA‐overview available on OSF: 10.17605/OSF.IO/G46ZR) revealed that a factor of similar latency, polarity and topography (Factor 4, peaking at 195 ms) was also part of that solution. It was less prominent, however, which is likely why we did not consider it as a key component for reporting. The positive difference in the P3a time range observed in the current study received contributions from two components identified as an early P3a (peak at 246 ms) and a late P3a (peak at 289 ms). This fits well with the characterisation of P3a in the literature as consisting of two subcomponents (Dien et al. 2004; Escera et al. 2000; Polich 2007). Despite notable differences in SOAs, the current EFA solution is similar to the one obtained in the preceding study (Coy et al. 2024) regarding the number of factors but also concerning their latency and topography (with exception of late P3a), indicating a reliable decomposition of the observed ERP into its underlying components.

### 4.3 First Deviants

When comparing first deviants against the global standard, the modulation of N1 and P2 corresponded to the observed negative difference identified as MMN. With regard to the conditional rules associated with the rarely encountered deviants, P2 amplitude did not differ as a function of deviant repetition probability. The early portion of the observed MMN, that is, the N1 amplitude, was descriptively slightly smaller for the high compared to the low repetition probability deviant, which would plausibly fit with the former deviant type occurring 50% more often. But unlike in the preceding study (Coy et al. 2024), Bayesian evidence is inconclusive. Although base rate affects N1 amplitude (Jääskeläinen et al. 2004), in the current study, the temporal separation between sounds was longer than in Coy et al. (2024), such that N1 adaptation effects would reasonably be smaller (May 2021). First deviants relative to the global standard elicited P3a activity of similar magnitude irrespective of their repetition probability. This indicates that the global rule (*the most likely sound on any trial is the standard*) was successfully extracted by the auditory system.

### 4.4 Deviant After Deviant

Research Question 1 was whether the auditory system learns to predict a second deviant (based on conditional probability) even though this stimulus violates the global rule (defined by base probability: *The most likely sound on any trial is the standard*). Although there is evidence that this is the case at the level of behaviour during active listening, we were interested in whether this is also possible during passive listening. We follow the logic of MMN studies by probing whether there is a difference (i.e., MMN or P3a) in the contrast between violation and confirmation of deviant repetition rules. Thus, if the rules embedded in the rarely encountered deviants are extracted, in the case of a deviant following a deviant (deviant after deviant), there should be a difference in MMN (and/or in P3a) amplitude in the contrast between the deviant type associated with low repetition probability (violation of the repetition rule) and the deviant type associated with high repetition probability (confirmation of the repetition rule).

Visually, there is a small negative difference in the current study between conditionally unlikely (low probability of repetition) and likely (high probability of repetition) deviants at the level of the two P2 subcomponents (i.e., in the MMN time range), yet the Bayesian analysis indicates that evidence is inconclusive. In the preceding study (Coy et al. 2024), there was moderate evidence against a negative difference in the typical MMN time range, both at the level of N1 and P2 in this low‐minus‐high repetition probability contrast. In the time range of P3a, there was moderate evidence against a positive difference in the current study (early and late P3a), which is also what we found in the preceding study for early P3a (no late P3a component was observed in either condition there).

Thus, considering the findings from both studies, there is no substantial evidence that MMN appears to be elicited in response to the violation of a conditional repetition rule when in disagreement with the global rule (i.e., deviant‐after‐deviant sounds always violate the standard regularity because they are rare). This finding is in line with a study by Todd et al. (2010), finding no modulation of the change in MMN from first to second deviants as a function of the conditional probability (100% vs. 50% between blocks). However, Sussman and Winkler (2001) reported a modulation of MMN amplitude as a function of deviant repetition probability. Their study's design differs from the current study in several respects (e.g., rules change between not within blocks, faster stimulation speed), which—as we discussed in detail in the preceding study (Coy et al. 2024)—might explain the divergence in the obtained results.

### 4.5 Standard After Deviant

Research Question 2 was whether the auditory system can process a standard stimulus as a mismatch to a local rule (conditional probability) although the stimulus is in agreement with the global rule (base rate). Note that the classic oddball paradigm typically comprises only single deviants that are followed by a standard in 100% of the cases. The few studies that analysed these standards (Koistinen et al. 2012; Nousak et al. 1996; Roeber et al. 2003, 2005; Sams et al. 1984) did not include an appropriate control, because the standard after deviant was compared against the global standard. Here, when a first deviant was followed by a standard sound (standard after deviant), we contrasted high (rule violation) against low (rule confirmation) repetition probability of the preceding deviant. There was no indication of a negative difference in the MMN time range (i.e., N1 and P2) nor of a positive difference in the P3a time range (early and late P3a).

In the preceding study (Coy et al. 2024), we observed a positive‐going trend in the grand‐average for this contrast. Although evidence within the MMN time range was inconclusive, there was moderate evidence of P3a‐like activity in response to the unpredictable standard (high repetition probability) compared to the predictable standard (low repetition probability). In the current study, we did not observe an indication of P3a elicitation. This divergence is somewhat puzzling. One could imagine that it is due to the changes, we made in the temporal characteristics of the stimulation (here, we used a longer and fixed SOA of 800 ms, whereas in the preceding study, SOA was uniformly jittered between 550 and 700 ms). Temporal regularity has been suggested to benefit regularity extraction (Schwartze et al. 2011; Tavano et al. 2014). Thus, one would have expected that the temporally more regular structure employed in the current study (constant SOA) compared to the irregular structure of the preceding study (jittered SOA) would have promoted extraction of the deviant rules. Yet it is also conceivable that the shorter and variable SOA in the preceding study (Coy et al. 2024) has resulted in varying overlap between consecutive trials, such that the processing of a preceding sound was still ongoing when the current sound was presented. Due to the variable SOA, more or less ‘spillage’ of processing related to the preceding sound into the current trial may have occurred, which, especially in cells with a lower number of trials, could have introduced more noise but could potentially also have interacted with the processing of the current sound. Apart from this, it has been shown that stimulation speed affects how regularities are represented (Müller et al. 2005a; Müller and Schröger 2007; Tervaniemi et al. 1999), therefore extracting rarely occurring relationships between sounds might simply be more difficult when their temporal separation increases.

Another potential explanation for the divergence at the level of early P3a is related to variability between listeners. We conducted post hoc exploratory analyses that can be found in Part B of the Supporting Information. When drawing random subsamples from the full sample (10,000 draws with 40 randomly selected [out of the 60] participants), it appears that the effect of conditional rules both in terms of statistical evidence and magnitude is relatively consistent between listeners when the sound following a first deviant violates the global rule (deviant‐after‐deviant), whereas there is considerable variability between subsamples when that sound conforms to the global rule (standard after deviant). Furthermore, the range of obtained effect sizes (Figure S2) indicates that even if there are true effects related to the processing of rarely encountered conditional rule violations, they are very small and substantially larger sample sizes would be required to reliably detect them.

### 4.6 Frequency of Rule Encounters: Global Versus Conditional Rule

It has been shown that the auditory system is sensitive to complex rules governing the transition between sounds (Bendixen et al. 2008, 2015; Saarinen et al. 1992; Tervaniemi et al. 1999; Tsogli et al. 2019, 2022). However, these findings relate to situations where it is the standard regularity that is defined by conditional probabilities (also referred to as transitional probabilities) between different sounds. In contrast, in the current study, the conditional rules are encountered far less often because they are associated with sounds that occur rarely among several other events that conform to another rule (i.e., the standard regularity). Therefore, in our stimulation protocol, we have two types of rules that can be used to predict the sound following a deviant: the conditional rules (defined by the conditional probability of repetition) and the global rule (defined by base rate: standards are frequent, and deviants violate the standard regularity because they are rare). Notably, base rate does not necessarily rely on the global probability of the stimulus itself but could also be considered in terms of transitions: Standard‐to‐standard transitions occur frequently, deviant‐to‐*x* (*x* either being a standard or a deviant) transitions occur rarely (Schröger et al. 2023; Winkler 2007). We have already argued previously that at least at the sensory processing level, it is possible that a rule needs to be encountered frequently in order to inform processing (Coy et al. 2024). However, in the preceding study, we did not conduct a comparison between standards and deviants following a first deviant, because, due to the notable drift in standard‐after‐deviant stimuli, baseline correction heavily affected the contrast against the deviant‐after‐deviant stimuli. The longer SOA of the current study rectified the ERP overlap and thereby improved baseline estimation and correction, such that we can now substantiate this hypothesis through a formal test: We observed very strong evidence that variance in the selected ERP components (in the time range of MMN and early P3a) is explained by the frequently encountered global rule but moderate evidence against a role of the rarely encountered conditional rules. From such moderate Bayesian evidence in favour of the null hypothesis, one should not infer that rarely encountered (conditional) rules are definitely not extracted by the auditory system. We did observe a small descriptive effect of the conditional rules on the processing of deviant‐after‐deviant stimuli, although statistical evidence did not substantiate this. Yet, to achieve an 80% probability of detecting a true effect with a *BF*_10_ evidence threshold of 6 (moderate evidence) when the population effect is 0.2, as observed in the current study, a sample size of 330 participants would be required. That is, even if there is an effect, it is certainly small and does not outweigh the considerable influence of the frequently encountered (global) rule. It remains to be investigated why the polarity of the deviant‐standard difference in Position 2 at the level of early P3a was opposite to what is usually observed (and replicated here) for Position 1. But this does not affect our general conclusion that global probabilities outweigh rarely encountered conditional rules.

### 4.7 Theoretical Implications

The internal representations of auditory regularities built by the human auditory system have been conceived as internal or generative models (Friston 2005; Garrido et al. 2009; Winkler 2007; Winkler and Czigler 2012). These models are presumably validated constantly, by comparing predictions inferred from these models against the actually received (sensory) input. The difference between prediction and input is referred to as prediction error, and when brain signatures of prediction error can be observed, this is interpreted as an indication that a given regularity was successfully extracted (Näätänen and Winkler 1999). What is less well understood though is the question of what information is used to build internal models and drives prediction (Schröger et al. 2023). As internal models are often assumed to be hierarchical, it is possible that different types of information inform different processing levels. This is also important with regard to theory building for brain signatures of prediction error such as MMN: which information can and cannot modulate a given prediction error signal hopefully improves our understanding of the mechanisms underlying its elicitation.

The findings in the current and the preceding (Coy et al. 2024) study indicate that the frequency of rule encounters (global probability of a specific sound or transition between sounds) is a crucial factor determining auditory prediction as reflected by ERP markers such as MMN and P3a. In a passive‐listening context, prediction in the oddball paradigm appears to be driven by the standard regularity (global rule: *Standards are the most likely sound on any trial because they are frequent*). Importantly, other studies have demonstrated that even at sensory levels, standard regularities are extracted that are more complex than a mere repetition of the same sound, such as the repetition of transitions between sounds (for review, Paavilainen 2013). Thus, in a more general sense, one could posit that auditory sensory prediction operates on the following heuristic: *What has been the case often in the recent past is likely to be the case in the immediate future*. This conception of prediction aligns well with the conception of MMN as a reflection of a (likely asymptotic) change in neural responsiveness to the standard as a function of the number of preceding rule‐conforming events (Baldeweg 2006; Baldeweg et al. 2004; Bendixen et al. 2008; Friston 2005; Haenschel et al. 2005). Furthermore, this heuristic supports adaptation as a prime candidate for the underlying neural mechanism, encoding recurrent sensory information both at the level of single sounds and at the level of between‐sound transitions (Bendixen et al. 2008; Garrido et al. 2009; May and Tiitinen 2010; Schröger et al. 2023; Ulanovsky et al. 2003). At higher processing levels, the available findings paint a less clear picture. Although, in our preceding study (Coy et al. 2024), we found evidence suggesting that these higher levels (indicated by P3a) are sensitive to rarely encountered rules, the findings in the current study do not confirm this observation. We do not have a clear explanation for this discrepancy, except for a potential role of the temporal separation between sounds and consequently respective deviant events (i.e., longer SOAs might impede rule extraction) and some tentative indications of interindividual differences in the extraction of rarely encountered rules during passive listening (see Supporting Information Part B)—this might be an interesting focus for future research.

Apart from this, there is a curious divergence between electrophysiological indicators of rule extraction during passive listening and the prominent behavioural effects during active listening. The gradual emergence of the response time differentiation as a function of the conditional rules associated with the deviants suggests that some time is required before these rules measurably affect behaviour. However, we do not know whether the preceding passive‐listening task might have had a beneficial effect, such that the conditional deviant repetition rules were extracted faster than had there been no prior exposure. Behavioural relevance of certain rules can arguably facilitate their extraction (e.g., Bendixen and Schröger 2008; Huettel et al. 2002; Rüsseler and Rösler 2000). However, even when participants are able to detect violations of a (standard) regularity based on contingencies between different sounds, they are not necessarily able to report what the rule is (van Zuijen et al. 2006). Furthermore, for sequence learning it has been shown that some participants rely on contingencies between responses rather than between stimuli (Rüsseler and Rösler 2000). Thus, it is possible that the conditional rules associated with the rarely encountered deviants in the current study might be acquired during active listening through stimulus–response learning rather than sensory learning. Nonetheless, according to predictive coding, the relative influence of prediction error can be modulated according to top‐down prior expectations (Garrido et al. 2009). This raises the question whether the strength of prediction might also be modulated by current or previous goals. In a passive‐listening context without any prior exposure, it may not be efficient or even necessary to top‐down project predictions related to rare events in sensory areas. Thus, such representations may exist but may be too weak or too unstable, or their precision may be too low during passive listening, to exert a measurable effect on the ERP components typically considered to reflect auditory prediction error (viz., MMN and P3a). As it has been shown that the initial context in which sounds are encountered can shape later processing of them even when the context changes (Fitzgerald and Todd 2020), it seems possible that rarely encountered conditional rules associated with deviants inform processing after all during passive listening when these rules were relevant in a preceding task. Although it is generally assumed that model maintenance as indexed by MMN elicitation does not require focused attention (Garrido et al. 2009; Näätänen et al. 2007; Sussman 2007; Sussman et al. 2014), it would be interesting to obtain electrophysiological indicators during or even after active listening, to see whether focused attention, task relevance or prior experience can modulate model maintenance and inference, as has been demonstrated for standard regularities defined by complex sounds (Näätänen et al. 1993). Furthermore, it should be noted that ERP markers (such as MMN and P3a) are popular tools to investigate prediction but they certainly are not the only ones. It is conceivable that rarely encountered rules, such as those employed in our modified oddball design, are represented differently than rules that are encountered frequently. Future studies may tap into other neural signatures such as oscillatory dynamics, which may provide further insights.

## 5 Conclusion

To conclude, our findings confirm that rarely encountered rules (based on conditional probability) can be extracted when directly behaviourally relevant. Electrophysiological indicators (MMN and P3a) do not appear to (reliably) reflect predictive conditional probability information when embedded in rarely encountered events. In fact, processing during passive listening as reflected in ERP components such as MMN and P3a appears to be foremost driven by the frequency with which rule‐conforming events occur (in the classic oddball with only one rule, this corresponds to global stimulus probability). Thus, it appears that deviants may be considered both a failure of prediction (related to the global standard regularity) and, at least under certain conditions, a driver of prediction. However, further investigation is required to elucidate the influence of contextual parameters such as attention and behavioural goals on electrophysiological processing of rarely encountered (conditional) rules associated with deviants. Disentangling the type of information (conditional vs. global stimulus probability) from the frequency of rule encounters (rare vs. frequent) provides an additional approach to improve our understanding of how our brains cope with the complexity and dynamics of the world.

## Author Contributions

**Nina Coy:** conceptualization, data curation, formal analysis, investigation, methodology, project administration, software, visualization, writing – original draft, writing – review and editing. **Alexandra Bendixen:** conceptualization, methodology, supervision, writing – review and editing. **Annika Löhr:** conceptualization, investigation, software, visualization, writing – review and editing. **Sabine Grimm:** conceptualization, supervision, writing – review and editing. **Erich Schröger:** conceptualization, resources, supervision, writing – review and editing. **Urte Roeber:** conceptualization, project administration, supervision, writing – review and editing.

## Funding

This study was funded by the Bundesministerium für Bildung und Forschung (www.bmbf.de) with Funding Number M526300. The funder had no role in study design, data collection and analysis, decision to publish or preparation of the manuscript.

## Ethics Statement

Protocols and procedures were in accordance with the Declaration of Helsinki and approved by the Ethics Advisory Board at Leipzig University (RF: 2022.02.22_eb_134).

## Conflicts of Interest

The authors declare no conflicts of interest.

## Supporting information


**Figure S1:** Posterior predictive for Bayesian multilevel model of single‐trial response times including time as an additional predictor. We used 100 draws from the posterior distribution. Each dot represents a single observed response. The shaded areas represent, from dark to light shade, the 50th, 80th, 95th and 99th percentile of the posterior predictive. The thick solid line represents the expected value (median) as a function of time (actually, the trial number of a given deviant sound within the whole behavioural part).
**Figure S2:** Ridgeline plots of random subsample e:ect size estimates and Bayes Factors. We used 10,000 subsample random draws (Nsub = 40) from the full sample (Nfull = 60). Note that the Bayes Factor is on the log‐scale: values below 0 favour the null hypothesis, values above 0 the alternative. Respective estimates based on the whole sample are marked by the circular (Coy et al. 2024) and triangular (current study) shapes. Densities are estimated using a Gaussian kernel density estimator and a Silverman bandwidth.

## Data Availability

The data that support the findings of this study and the scripts used for statistical analysis are openly available in the OSF Repository at https://doi.org/10.17605/OSF.IO/FG9HN.
