# Supplementary material for: Not All Rules Are Equal: Rare Conditional Rules Shape Behaviour but Yield to Global Probability in Passive Listening
Source: Eur J Neurosci. 2026 Apr 20;63(8):e70500. doi: 10.1111/ejn.70500 (PMC13096706; doi:10.1111/ejn.70500)
Supplement: Supplementary file 1 — Figure S1: Posterior predictive for Bayesian multilevel model of single‐trial response times including time as an additional predictor. We used 100 draws from the posterior distribution. Each dot represents a single observed response. The shaded areas represent, from dark to light shade, the 50th, 80th, 95th and 99th percentile of the posterior predictive. The thick solid line represents the expected value (median) as a function of time (actually, the trial number of a given deviant sound within the whole behavioural part). Figure S2: Ridgeline plots of random subsample e:ect size estimates and Bayes Factors. We used 10,000 subsample random draws (Nsub = 40) from the full sample (Nfull = 60). Note that the Bayes Factor is on the log‐scale: values below 0 favour the null hypothesis, values above 0 the alternative. Respective estimates based on the whole sample are marked by the circular (Coy et al. 2024) and triangular (current study) shapes. Densities are estimated using a Gaussian kernel density estimator and a Silverman bandwidth. [file EJN-63-0-s001.pdf]

Not all rules are equal: rare conditional rules shape behaviour but yield to global probability in passive listening

Nina Coy, Alexandra Bendixen, Annika Löhr, Sabine Grimm, Erich Schröger and Urte Roeber

Supplemental material

This supplement consists of two parts: Part (A) gives an additional plot of the single-trial analysis of response time data to provide a more detailed overview of the model fit. Part (B) shows a post-hoc exploration of potential inter-individual differences.

Part A: Single-trial response time analysis

Figure S1 depicts probability bands obtained from the posterior predictive (based on 100 draws from the posterior distribution) as a function of time (i.e., the trial number at which a given deviant sound occurs). The posterior predictive relates to the probability of single observations given the posterior distribution of parameters. As the vast majority of actual observations (single dots in Figure S1) fall inside the probability ribbons marking up to the 99th percentile of the posterior predictive, using a lognormal link function the chosen model appears to provide a relatively good fit not only at the level of the central tendency (here, median) but also at the level of single observations. There is a slight tendency to underestimate extreme values (especially long RTs). Nonetheless, the skewed layering of the probability bands towards earlier RTs and around the expected value (central tendency: median) indicates that the model captures the skew in the RT distribution quite well. A more detailed overview of model fit and statistics can be found on OSF in the respective markdown document (statistical analysis of behavioural data).

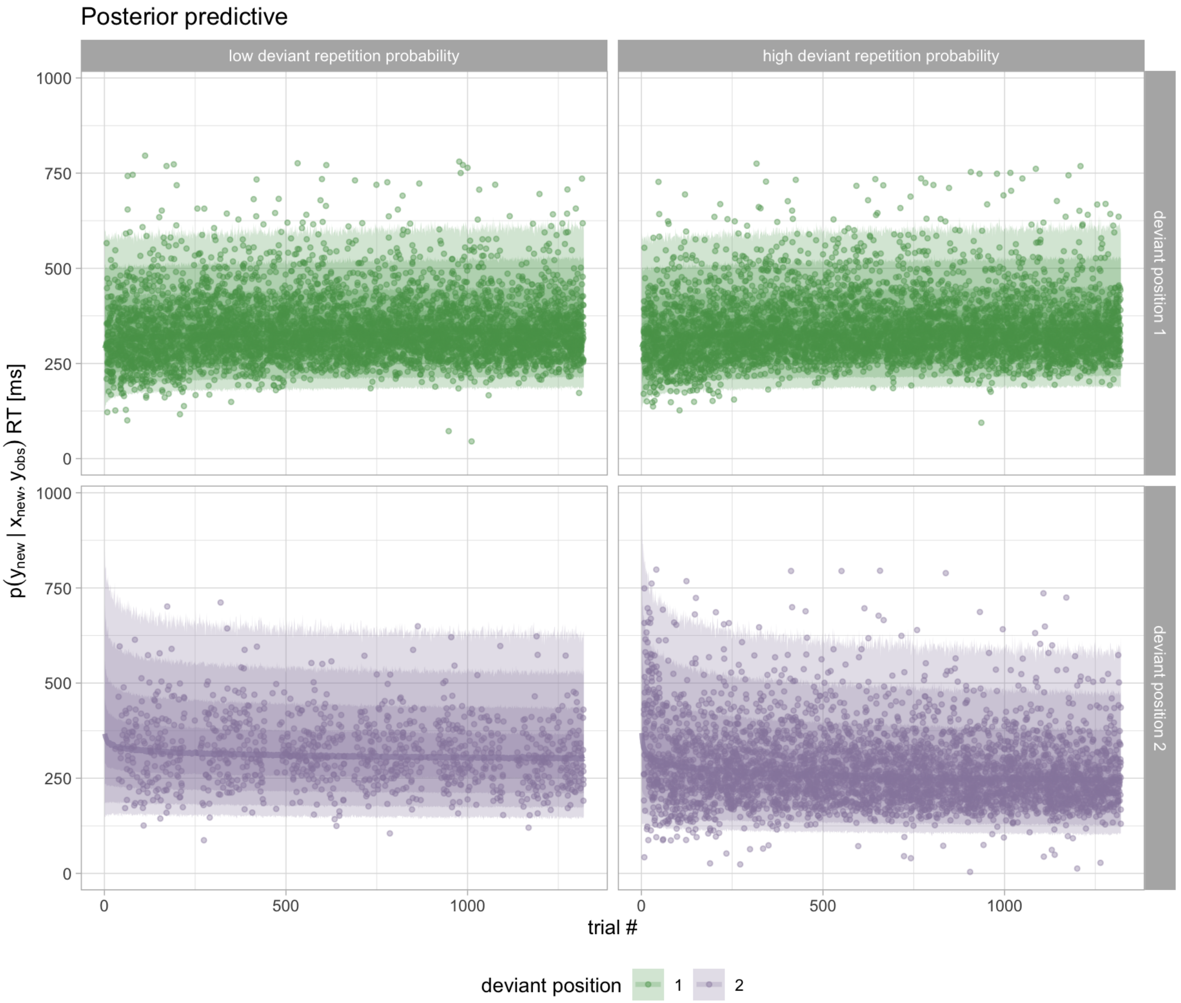

Figure S1. **Posterior predictive for Bayesian multilevel model of single-trial response times including time as an additional predictor.** We used 100 draws from the posterior distribution. Each dot represents a single observed response. The shaded areas represent, from dark to light shade, the 50th, 80th, 95th and 99th percentile of the posterior predictive. The thick solid line represents the expected value (median) as a function of time (actually, the trial number of a given deviant sound within the whole behavioural part).

Part B: Divergence between studies regarding the extraction of rarely encountered rules

In our previous study (Coy et al., 2024) we found moderate evidence in favour of (early) P3a elicitation for conditional rule violations in standard stimuli (though not for deviant stimuli). In contrast, in the current study we found no evidence suggesting P3a elicitation. Listeners might differ in their ability to extract rarely encountered rules (here, conditional repetition probability associated with deviant sounds) or in the way respective rule violations are processed (van Zuijen et al., 2006). For complex standard regularities, it has been shown that ERPs differ between participants that became aware of the nature of the regularity and participants that remained naïve (van Zuijen et al., 2006). Potential differences in the composition of the participant samples might thus explain the differing statistical evidence when comparing the averages between the studies. Nonetheless, it is also possible that there are more systematic differences between the two studies such as the temporal separation between stimuli: in the current study we used a longer and fixed SOA of 800 ms compared to the preceding study, in which SOA was uniformly jittered between 550-700 ms.

If inter-individual differences in the extraction and representation of rarely encountered rules mainly underlie the aforementioned divergence between the two studies, we would expect that the P3a effect depends on a particular composition of a sample. To explore this, for each study we drew 10,000 subsamples each consisting of 40 randomly selected participants from the respective whole sample (60 participants) and computed Bayes Factors (BF_{10}) and effect size estimates (Cohen's d_z) of the early P3a amplitude estimate.

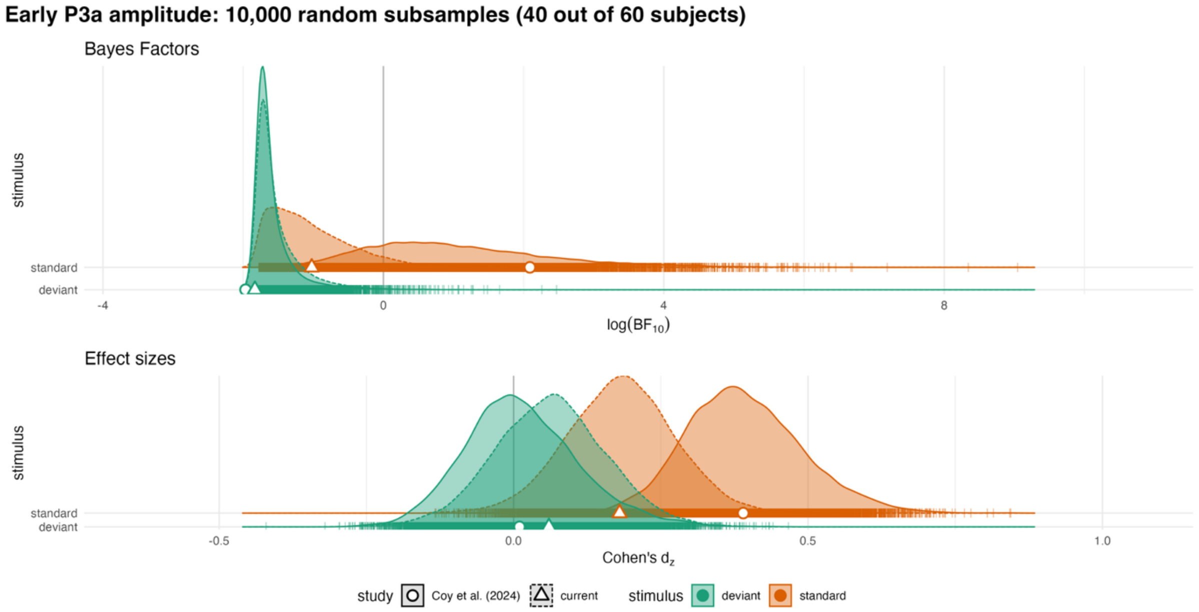

Figure S2. **Ridgeline plots of random subsample effect size estimates and Bayes Factors.** We used 10,000 subsample random draws (N_{sub}=40) from the full sample (N_{full} = 60). Note that the Bayes Factor is on the log-scale: values below 0 favour the null hypothesis, values above 0 the alternative. Respective estimates based on the whole sample are marked by the circular (Coy et al. 2024) and triangular (current study) shapes. Densities are estimated using a Gaussian kernel density estimator and a Silverman bandwidth.

The empirical effect size estimates all fall well into the range obtained from the subsamples (Figure S2 bottom). There appears to be some considerable variability between subsamples, such that the effect size is notably smaller or larger in some subsamples compared to when considering the whole sample. This indicates that there are some differences between participants, although visually the spread does not seem to differ between studies. For deviant-after-deviant stimuli the densities of subsample effect size estimates show a large overlap between studies; centred close to zero in both cases. Notably, for standard-after-deviant stimuli there is a clear separation of the effect size densities, with the preceding study shifted towards larger effect sizes. Nonetheless, in the current study the largest portion of the density spans values greater than zero but within the range of small effects.

When looking at the level of statistical evidence in the form of Bayes Factors (BF_{10}), there is a notable difference between conformation of stimuli to the global rule. In both studies most subsamples support the null hypothesis (i.e., no elicitation of early P3a in response to rarely encountered conditional rule violations) when the stimuli in question violate the global rule (deviant-after-deviants). The corresponding empirical Bayes Factors of each study (Figure S2 top; green shapes) are located outside the subsample range. Considering the high consistency between subsamples, this likely reflects greater accumulated evidence in the full sample.

When the stimuli in question conform to the global rule (standard-after-deviants), there is considerably more variability in the obtained Bayes Factors between subsamples, indicating less consistency in P3a effects between participants. Especially in the preceding study, *BF*s appear to depend heavily on the specific subsample.

The sample size for both studies was predetermined following the fixed-n approach for planning informative Bayes Factor (*BF*) designs (Schönbrodt et al., 2017), such that there is at least an 80% probability of detecting a true effect with a BF_{10} evidence threshold of 6 (moderate evidence) when the population effect is 0.5 (medium-sized). For the effect size actually obtained in the current study (approximately 0.2), even when applying an evidence threshold of 3, power was at 18%, and a sample size of 350 would have been necessary to achieve the desired level of power.

Taken together, this exploration via subsampling underlines that, even if there are true effects related to the processing of rarely encountered conditional rule violations, they are small and substantially bigger sample sizes would be required to reliably detect them.
